# Leptin increases mitochondrial OPA1 via GSK3-mediated OMA1 ubiquitination to enhance therapeutic effects of mesenchymal stem cell transplantation

**DOI:** 10.1038/s41419-018-0579-9

**Published:** 2018-05-10

**Authors:** Fan Yang, Rongrong Wu, Zhi Jiang, Jinghai Chen, Jinliang Nan, Sheng’an Su, Na Zhang, Chen Wang, Jing Zhao, Cheng Ni, Yingchao Wang, Wangxing Hu, Zhiru Zeng, Keyang Zhu, Xianbao Liu, Xinyang Hu, Wei Zhu, Hong Yu, Jinyu Huang, Jian’an Wang

**Affiliations:** 1 Department of Cardiology, Second Affiliated Hospital, Zhejiang University College of Medicine, Hangzhou, Zhejiang China; 2 Provincial Key Laboratory of Cardiovascular Research, Hangzhou, Zhejiang China; 3 Department of Cardiology, Guizhou Provincial People’s Hospital, Guizhou, China; 4 Institute of Translational Medicine, Zhejiang University Hangzhou, Zhejiang, China; 5 Department of Cardiology, Hangzhou First People’s Hospital, Hangzhou, China

## Abstract

Accumulating evidence revealed that mesenchymal stem cells (MSCs) confer cardioprotection against myocardial infarction (MI). However, the poor survival and engraftment rate of the transplanted cells limited their therapeutic efficacy in the heart. The enhanced leptin production associated with hypoxia preconditioning contributed to the improved MSCs survival. Mitochondrial integrity determines the cellular fate. Thus, we aimed to investigate whether leptin can enhance mitochondrial integrity of human MSCs (hMSCs) to protect against various stress. In vivo, transplantation of leptin-overexpressing hMSCs into the infarcted heart resulted in improved cell viability, leading to enhanced angiogenesis and cardiac function. In vitro, pretreatment of hMSCs with recombinant leptin (hMSCs-Lep^pre^) displayed improved cell survival against severe ischemic condition (glucose and serum deprivation under hypoxia), which was associated with increased mitochondrial fusion. Subsequently, Optic atrophy 1 (OPA1), a mitochondrial inner membrane protein that regulates fusion and cristae structure, was significantly elevated in the hMSCs-Lep^pre^ group, and the protection of leptin was abrogated by targeting OPA1 with a selective siRNA. Furthermore, OMA1, a mitochondrial protease that cleaves OPA1, decreased in a leptin-dependent manner. Pretreatment of cells with an inhibitor of the proteasome (MG132), prevented leptin-induced OMA1 degradation, implicating the ubiquitination/proteasome system as a part of the protective leptin pathway. In addition, GSK3 inhibitor (SB216763) was also involved in the degradation of OMA1. In conclusion, in the hostile microenvironment caused by MI, (a) leptin can maintain the mitochondrial integrity and prolong the survival of hMSCs; (b) leptin-mediated mitochondrial integrity requires phosphorylation of GSK3 as a prerequisite for ubiquitination-depended degradation of OMA1 and attenuation of long-OPA1 cleavage. Thus, leptin targeting the GSK3/OMA1/OPA1 signaling pathway can optimize hMSCs therapy for cardiovascular diseases such as MI.

## Introduction

The inherent properties include low immunogenicity^1^, multipotentiality, and maintenance of “stemness”^2^, deeming mesenchymal stem cells (MSCs) as the potential vectors of choice for regenerative medicine^3–5^. Pioneering studies by our group confirmed that hypoxia preconditioning could enhance the therapeutic efficiency of MSCs in rodent myocardial infarction (MI) models, and we further proposed that leptin was an obligatory intermediate in the anti-apoptotic properties of MSCs^6,7^.

Originally identified as a peptidase secreted by adipocytes, leptin plays a vital role in regulating metabolic normalization, neuroendocrine, and immune homeostasis^8–10^. Leptin improves hyperlipidemia through stimulation of lipid oxidation and restores glucose homeostasis via melioration of insulin resistance and suppression of hepatic gluconeogenesis^8,11^. Moreover, increasing studies demonstrated that inalienable relationships between metabolism and mitochondria are critical for cellular fate^12–14^. In addition, growing evidence suggested that the potential of leptin might contribute to mitochondrial changes. The disturbance of mitochondrial morphology results in obesity^15^. The exposure to leptin improves the mitochondrial function in MCF-7 cells and *ob/ob* mice^16,17^. In transverse aortic constriction models, mitochondria are regulated by STAT3, which is a canonical downstream intermediate of leptin signaling pathway^18^.

Mitochondrial integrity and morphology determine the cellular death and diseases by preventing the release of diverse pro-apoptotic factors, and excessive fragmentation of mitochondria promote cellular death^19–21^. Interestingly, mitochondrial fusion is a control point for apoptotic processes, and cellular death is tightly linked to mitochondrial dysfunction^21,22^. The opposite actions of mitochondrial proteins are responsible for healthy quality control, including Drp1 for fragmentation and Mfn1/Mfn2 for fusion in the outer mitochondrial membrane^22^. Optic atrophy 1 (OPA1) is responsible for the fusion and fission in the inner mitochondrial membrane (IMM) executed by different isoforms^22–24^.

In the present study, we demonstrated that leptin confers mitochondrial integrity of human MSCs (hMSCs) by potentiating the OPA1 accumulation. The pathway involves increased ubiquitination of OMA1, thereby modulating enhanced long-OPA1 isoforms (L-OPA1) and providing a novel therapeutic target via leptin/GSK3/OMA1/OPA1 axis.

## Results

### Leptin protects hMSCs against apoptosis in vivo and in vitro

The potential protective role of leptin for MSCs was shown in our previous study^7^. Herein, we determined whether leptin improvedhMSCs survival. Both in vivo and in vitro studies were described in Supplementary Figure S1. The characteristics of hMSCs were described in Supplementary Figure S2a. We also confirmed that the leptin receptors were expressed on hMSCs (Supplementary Figure S2b).

**Fig. 1:**
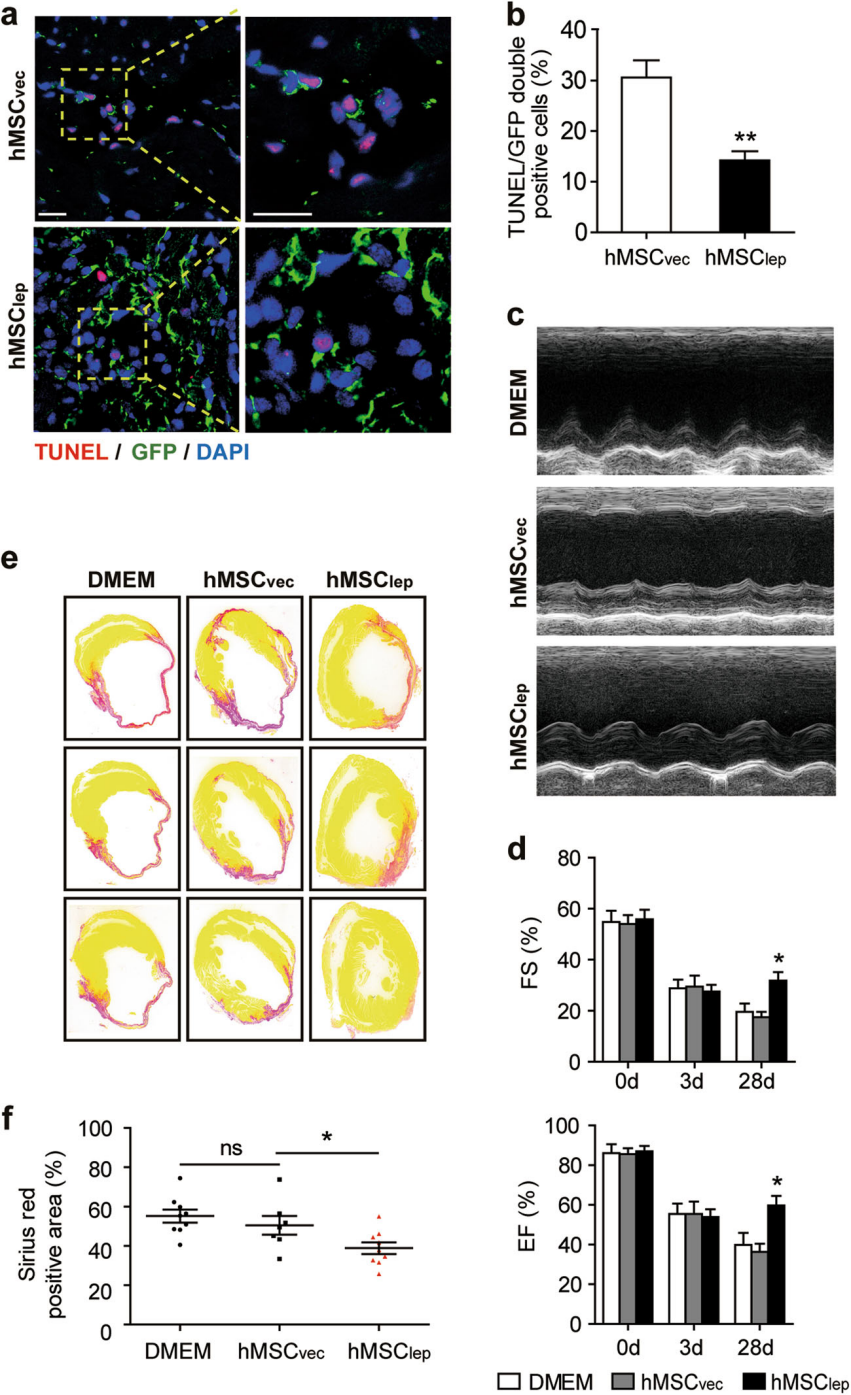
Leptin-mediated hMSCs treatment rescued cardiac function after MI in vivo. **a** Representative images of TUNEL staining assayed by confocal fluorescence microscopy, wherein apoptosis-positive cells (in red) were specifically counted for GFP-labeled hMSCs (in green); at least 200 GFP-labeled hMSCs were counted and analyzed for TUNEL staining for each heart sample, and five samples were analyzed for each group. Scale bar, 100 μm. **b** Apoptosis activity was expressed as the percentage of apoptotic hMSCs over the surviving hMSCs at day 3 post MI. **c** Representative echocardiographic image showed the changes in cardiac function for mice in each group at day 28 post MI. **d** Cardiac function including EF and FS were obtained from echocardiographic imaging (*n* = 5 for hMSC_vec_ group, *n* = 7 for DMEM, and hMSC_lep_ group, respectively). **e**, **f** At day 28 post MI, the infarction size was quantified by Sirius red staining expressed as the ratio of the length of collagen deposited area over the perimeter of the left ventricle with quantification shown in the bar graph (*n* = 7 for hMSC_vec_ group, *n* = 9 for DMEM, and hMSC_lep_ group, respectively). All data were expressed as mean ± SEM. *denotes *P* < 0.05. **P < 0.01

To confer the persistent expression of leptin in vivo, lentivirus containing a leptin-overexpression plasmid with green fluorescent protein (GFP) reporter was constructed and infected into hMSCs (hMSC_lep_), whereas the empty vector (hMSC_vec_) served as a control. The elevated expression level of leptin-induced by lentivirus infection of hMSCs was confirmed by western blot and immunofluorescence staining targeting leptin (Supplementary Figures S3a and S3b). An in vivo mouse MI model was employed for implanting the hMSC_lep_ into the peri-infarct zone immediately post surgery; equal numbers of hMSC_vec_ or an equal volume of Dulbecco's Modified Eagle Medium (DMEM) served as controls. A lower numbers of GFP-positive apoptotic cells were observed in the hMSC_lep_ group as compared with the relative to hMSC_vec_ group, confirmed by Terminal deoxynucleotidyl transferase dUTP nick end labeling (TUNEL) and GFP staining at day 3 post MI (*n* = 5 for each group; Figs. 1a, b). Two-dimensional echocardiography examination at day 28 post MI revealed that the hMSC_lep_ treatment group had a better cardiac function than either hMSC_vec_ or DMEM groups (*n* = 5 for the hMSC_vec_ group, *n* = 7 for DMEM and hMSC_lep_ group, respectively; Figs. 1c, d; Supplementary Table S1). These results were also consistent with the reduced infarct size measured by Sirius red staining at day 28 post MI, further supporting the enhanced therapeutic effects of transplanted hMSC_lep_ (*n* = 7 for hMSC_vec_ group, *n* = 9 for DMEM and hMSC_lep_ group, respectively; Figs. 1e, f).

**Fig. 2:**
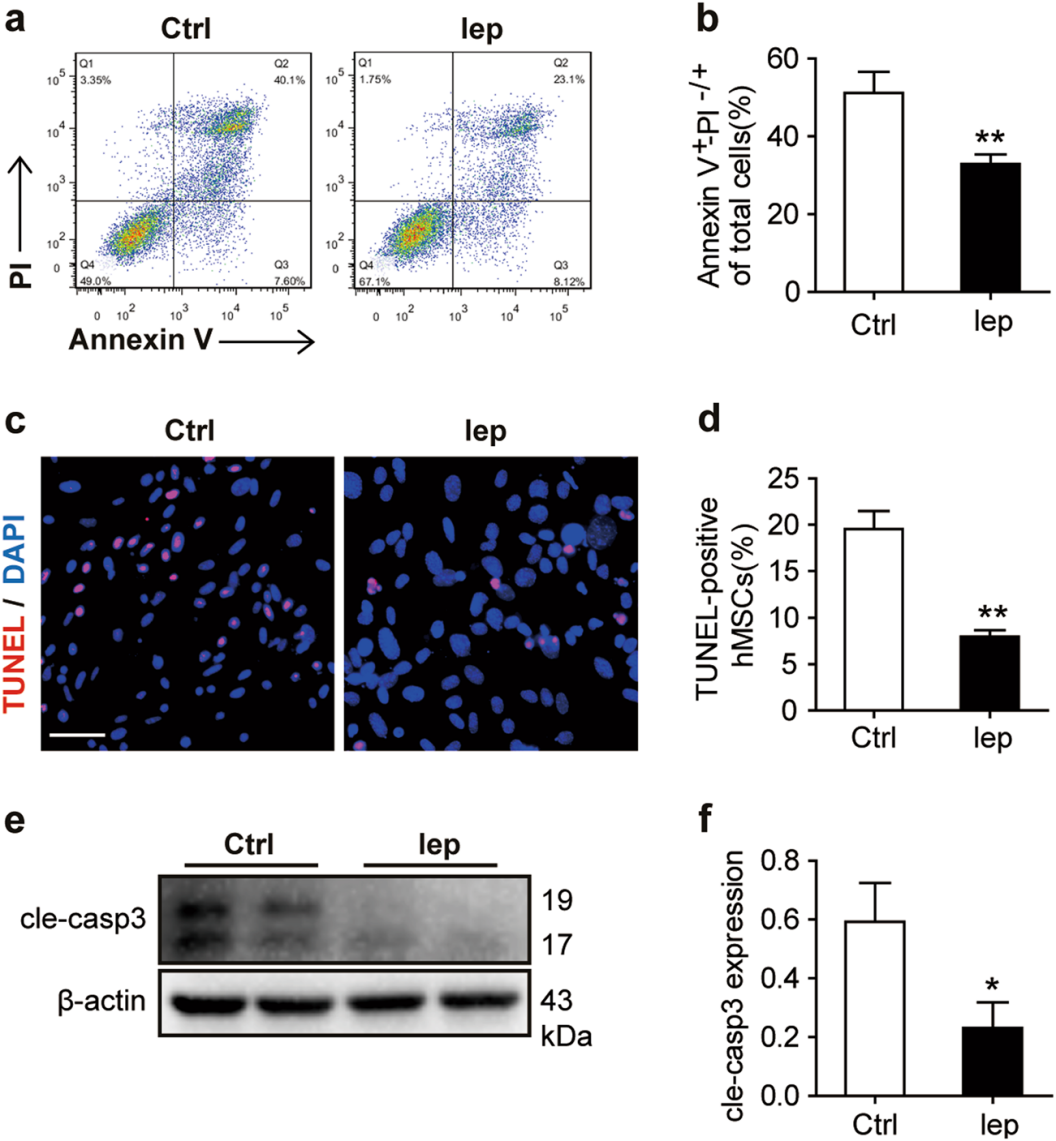
Leptin acted as a protective response for hMSCs against apoptosis in vitro. **a** Annexin V/PI staining was analyzed by FACS for cell apoptosis and necrosis. **b** Quantification of early (Q3) and late (Q2) apoptosis events were simultaneously measured. **c**, **d** Representative views of TUNEL staining with nuclei identified by DAPI staining (Scale bar, 50 μm) and apoptotic cells were quantified for TUNEL-positive nuclei over the total nuclei counted. The images were obtained from 8 to 10 randomly selected fields in each sample or per well. **e** Cleaved caspase 3 (cle-caspase 3) protein expression level of whole-cell protein lysates (WCL) was measured by western blot, and β-actin served as a loading control. **f** Protein expression levels were quantified by densitometry analysis. Each in vitro experiment was repeated three times. Data were shown as mean ± SEM. *denotes *P* < 0.05, **P < 0.01

For better understanding the physiological signaling of leptin activation, we pretreated the hMSCs with leptin in the in vitro assay. Therefore, we used the recombinant leptin (50 ng/ml)-pretreated hMSCs for 24 h under normoxic conditions (hMSCs-Lep^pre^), followed by glucose and serum deprivation under hypoxia (GSDH) stress for an additional 24 h (an ischemic+microenvironment mimic); Tris buffer (the solvent of leptin) was used as control (hMSCs-Ctrl^pre^). Reduced apoptosis was observed in the hMSCs-Lep^pre^ group as assayed by Annexin V/PI (propidium iodide) staining (Figs. 2a, b) and TUNEL staining (Figs. 2c, d) as compared with the hMSCs-Ctrl^pre^ group. Furthermore, the lower levels of cleaved caspase 3 also confirmed the reduced apoptosis in hMSCs-Lep^pre^ as compared with hMSCs-Ctrl^pre^ (Figs. 2e, 2f). To evaluate the efficiency of leptin on hMSCs by its gene overexpression in vitro (hMSC_lep_ or hMSC_vec_), after 48-h infection by lentivirus, these cells were exposed to GSDH for another 24 h. Consequently, decreased apoptosis rate was detected in hMSC_lep_ as compared to hMSC_vec_ as evidenced by Annexin V/PI staining (Supplementary Figures S4a and S4b), TUNEL staining (Supplementary Figures S4c and S4d), and low levels of cleaved caspase 3 (Supplementary Figures S4e and S4f). In addition, we further explored the efficiency of leptin on hMSCs under oxidative stress (H_2_O_2_) to mimic the microenvironment of MI by another model in vitro. After pretreatment with leptin (50 ng/ml) or solvent control for 24 h, the hMSCs were subjected to H_2_O_2_ (500 μM) treatment for 4 h. Similarly, the cardioprotection of leptin attenuated the apoptotic rate of hMSCs as detected by reduced Annexin V/PI staining (Supplementary Figures S5a and S5b), TUNEL staining (Supplementary Figures S5c and S5d), and levels of cleaved caspase 3 (Supplementary Figures S5e and S5f) in hMSCs-Lep^pre^ as compared with hMSCs-Ctrl^pre^. Together, leptin granted protection to hMSCs from ischemic insults.

### Leptin enhances paracrine efficacy of surviving hMSCs to induce angiogenesis

**Fig. 3:**
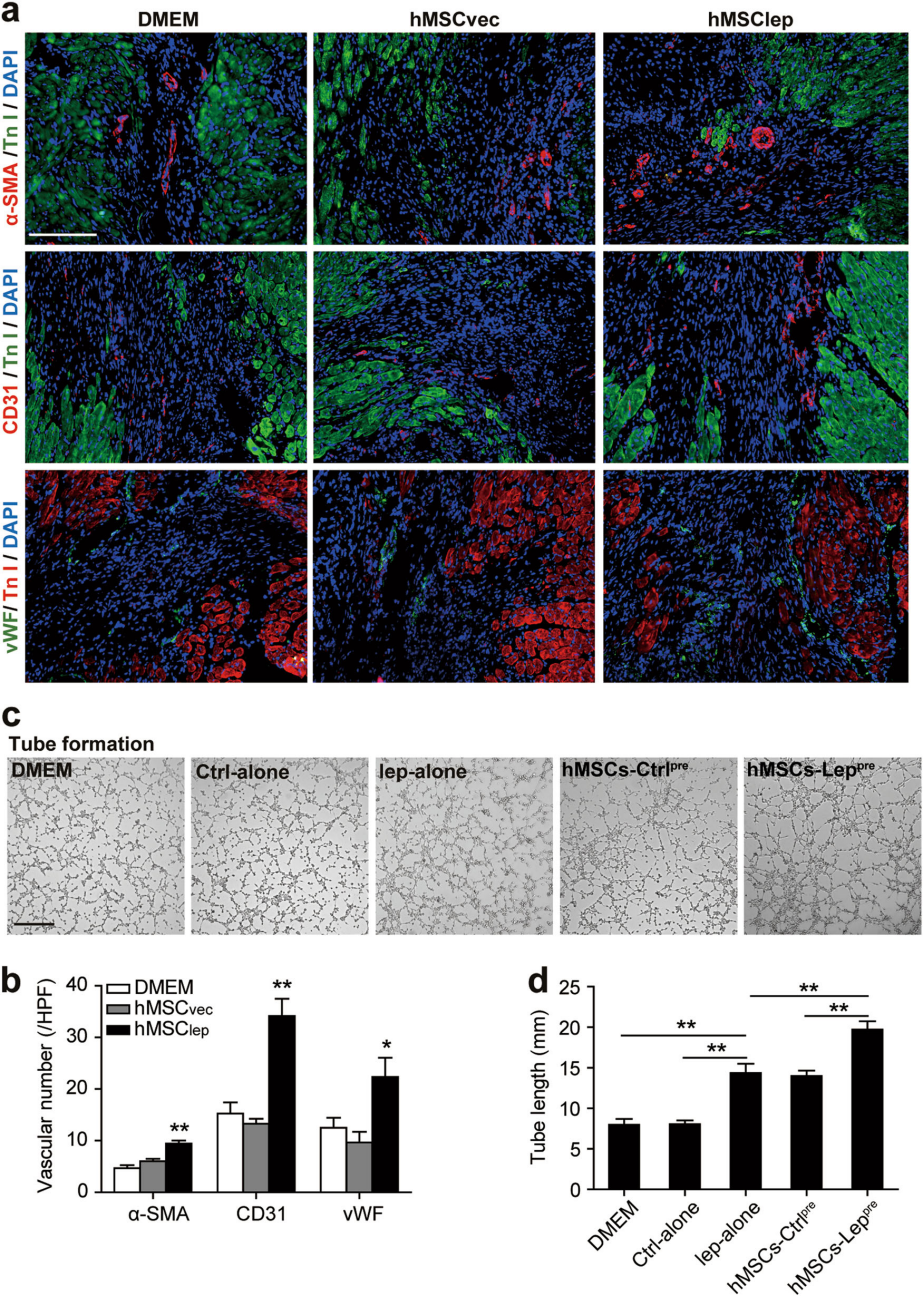
Paracrine efficacy of leptin-enhanced hMSCs improved angiogenesis. **a**, **b** Representative immunoflourescence of α-SMA, CD31, and vWF in the peri-infarct zone of ischemic hearts using heart tissue slides obtained from DMEM, hMSC_vec_, and hMSC_lep_ group mice (*n* = 7 for hMSC_lep_ group, *n* = 5 for DMEM, and hMSC_vec_ group each) at day 28 post MI and angiogenesis process was quantified by 6–8 high-power field (HPF) per section in the bar graphs. Scale bar, 100 μm. **c**, **d** Tube formation assay was conducted using HUVECs cultured with conditioned medium obtained from DMEM alone, control-alone, leptin alone, hMSCs-Ctrl^pre^, or hMSCs-Lep^pre^. The conditioned medium had been normalized by an equivalent number of hMSCs (1 × 10^6^ cells). The quantification of tube formation was shown in bar graphs. Scale bar, 50 μm. Independent in vitro experiment was repeated three times. Data were shown as mean ± SEM. *denotes *P* < 0.05, **P < 0.01

Increasing studies showed that the therapeutic effects of MSCs are primarily contributed by paracrine function^25–27^. Previously, our group found that hypoxia-preconditioned MSCs enhanced angiogenesis in MI-nonhuman primate model^28^. Regarding the paracrine mechanism of leptin-mediated protection of hMSCs in vivo, we observed an increased number of vascular smooth muscle cells and vascular endothelial cells in the peri-infarct area in hMSC_lep_ as compared with hMSC_vec_ or DMEM groups as assayed by the immunofluorescence staining of α-SMA, CD3, and vWF (Figs. 3a, b) at day 28 post MI. Furthermore, to investigate the paracrine mechanism of leptin-modulated hMSCs in vitro, the tube formation of human umbilical vein endothelial cells (HUVECs) in Matrigel assay demonstrated a significantly increased endothelial tube formation of HUVECs in conditioned medium of hMSCs-Lep^pre^ groups as compared with hMSCs-Ctrl^pre^ or leptin alone but not in DMEM alone and control-alone groups, whereas no statistical difference was detected in the tube formation in hMSCs-Ctrl^pre^ and leptin alone groups (Figs. 3c, d). These phenomena suggested that the beneficial effects of leptin on surviving hMSCs were enhanced through paracrine pathways, consistent with that observed previously^27,29,30^.

### Leptin induces fusion of mitochondrial networks in hypoxic hMSCs

**Fig. 4:**
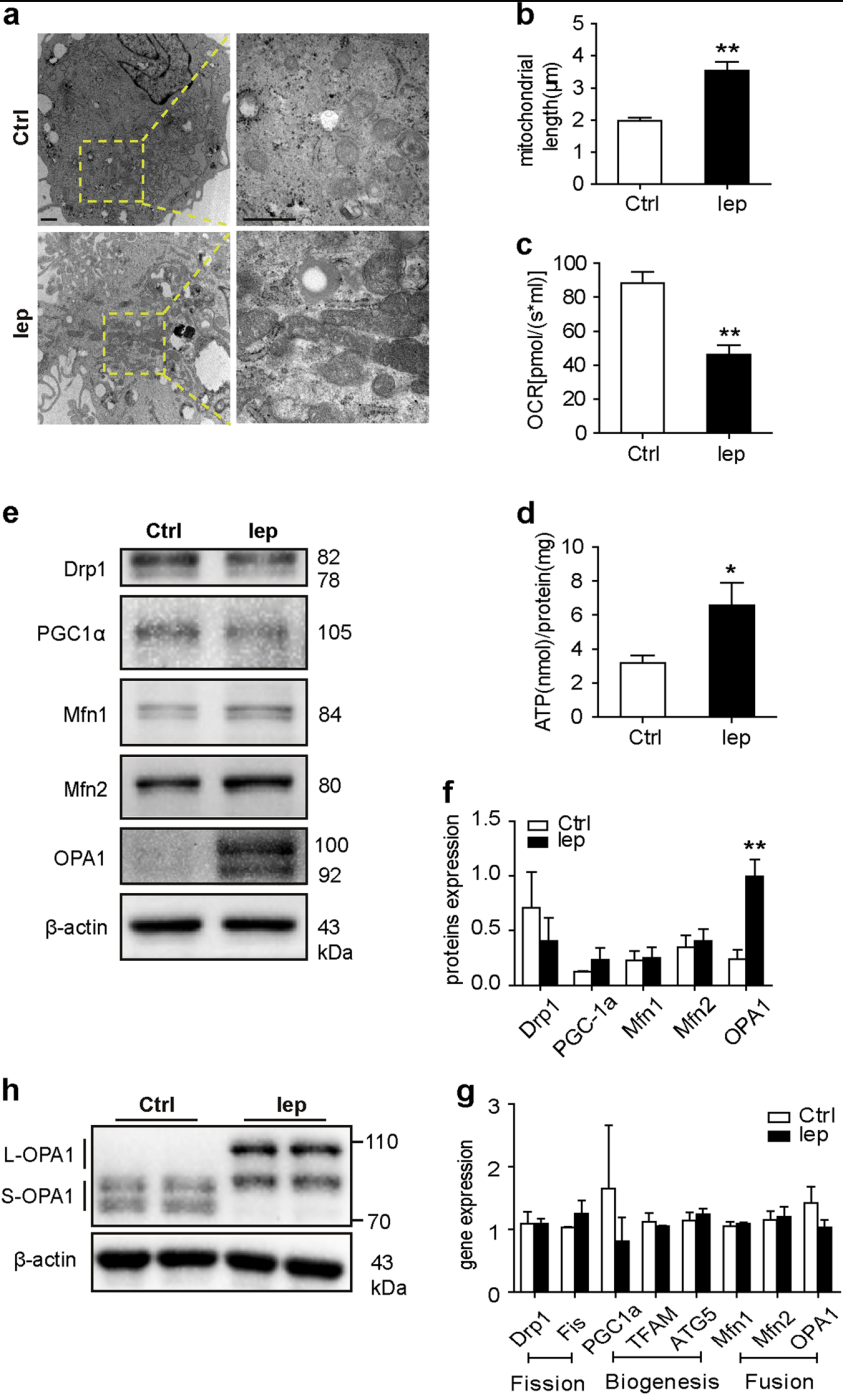
Leptin provoked fused mitochondrial network under GSDH condition. **a** Mitochondrial ultrastructures were analyzed by electron micrograph with representative images showing significant changes in mitochondrial length after hMSCs were pretreated with leptin (50 ng/ml) as compared with those pretreated with the solvent alone (magnification was set at × 15,000 and × 50,000, respectively); scale bar, 1 μm. **b** The mitochondrial length was measured using Adobe Photoshop CS5 for at least 20 mitochondria for each cell (at least 30 cells for each group). **c** OCR was quantified for both hMSCs-Lep^pre^ and hMSCs-Ctrl^pre^ using OROBOROS instrument. **d** Cellular ATP levels were conducted through luciferin/luciferase-based assay, and the data were calibrated with protein content. **e**, **f** Protein expression implicated mitochondrial homeostasis including fusion, fission, and biogenesis, and was assessed by western blot for hMSCs-Lep^pre^ and hMSCs-Ctrl^pre^ and quantified by densitometry using β-actin as loading control. **g** Expression levels of genes involved in mitochondrial homeostasis were detected with qRT-PCR normalized to that of β-actin. **h** The differential expression levels of L-OPA1 and S-OPA1 isoforms can be identified by western blot using the specific antibody for hMSCs-Lep^pre^ or hMSCs-Ctrl^pre^ in GSDH conditions for 24 h. Each experiment was repeated three times. All data were shown as mean ± SEM. *denotes *P* < 0.05, **P < 0.01

Several studies supported the key roles for mitochondrial dynamics in maintaining the normal mitochondrial morphology and function^19–21^. Healthy mitochondria undergo continuous fusion and fission processes to maintain normal function. To determine whether leptin regulates changes in mitochondrial morphology in hMSCs during ischemic stress, we analyzed the mitochondrial ultrastructure using transmission electron microscopy (TEM). hMSCs-Lep^pre^ cultured under the GSDH demonstrated dense and elongated tubular mitochondria with swollen cristae dispersed within the cells, whereas sparse, fewer, and punctate mitochondria were observed in hMSCs-Ctrl^pre^ (Fig. 4a). The mitochondria were also longer in hMSCs-Lep^pre^ relative to hMSCs-Ctrl^pre^ (Fig. 4b). However, no significant influence of leptin was exerted on the mitochondrial structure under normoxic culture conditions (Supplementary Figures S6a and S6b). Intriguingly, a lower oxygen consumption rate (OCR) was found when the mitochondrial respiratory function was measured in the hMSCs-Lep^pre^ as compared with hMSCs-Ctrl^pre^ group (Fig. 4c). These data strongly suggested that leptin promoted mitochondrial fusion to maintain the integrity, and this was unrelated with improvement of mitochondrial respiratory function.

Furthermore, we observed a remarkable ATP production in the hMSCs-Lep^pre^ group (Fig. 4d). Subsequently, both hMSCs-Lep^pre^ and hMSCs-Ctrl^pre^ groups displayed decreased mitochondrial membrane potential (ψMt) as measured by the Tetramethylrhodamine methyl ester (TMRM) indicator. Compared with hMSCs under normal culture conditions, the ψMt was ameliorated in hMSCs-Lep^pre^ group (Supplementary Figures S6c and S6d).

Next, we tested several pivotal proteins that were involved in the mitochondrial homeostasis. In contrast to hMSCs-Ctrl^pre^, the fusion regulator OPA1 (100, 92 kDa) showed enhanced expression in hMSCs-Lep^pre^, whereas some other well-known proteins involved in the mitochondrial dynamics did not alter significantly (Figs. 4e and 4f). In addition, qRT-PCR did not show any significant change in the mRNA levels, indicating that leptin did not affect the transcriptional activity of OPA1 and other regulators (Fig. 4g).

Moreover, different OPA1 isoforms (in the range of ~75–100 kDa) are known to perform counter-regulatory roles in maintaining mitochondrial dynamics, and L-OPA1 (~85–100 kDa) regulate the mitochondrial fusion, whereas short-OPA1 isoforms (S-OPA1, ~75–85 kDa) are correlated with mitochondrial fission^15,24^. Therefore, we further dissected the specific OPA1 isoforms using poly-OPA1 antibody and found strikingly different patterns of OPA1 expression in hMSCs-Lep^pre^ relative to hMSCs-Ctrl^pre^. Also, abundant L-OPA1 expression was observed in the hMSCs-Lep^pre^ group (Fig. 4h), whereas S-OPA1 was higher in the hMSCs-Ctrl^pre^ group. These differences might contribute to the enhanced mitochondrial fusion processes described above.

### OPA1 is required for leptin-augmented hMSCs survival under ischemic conditions

**Fig. 5:**
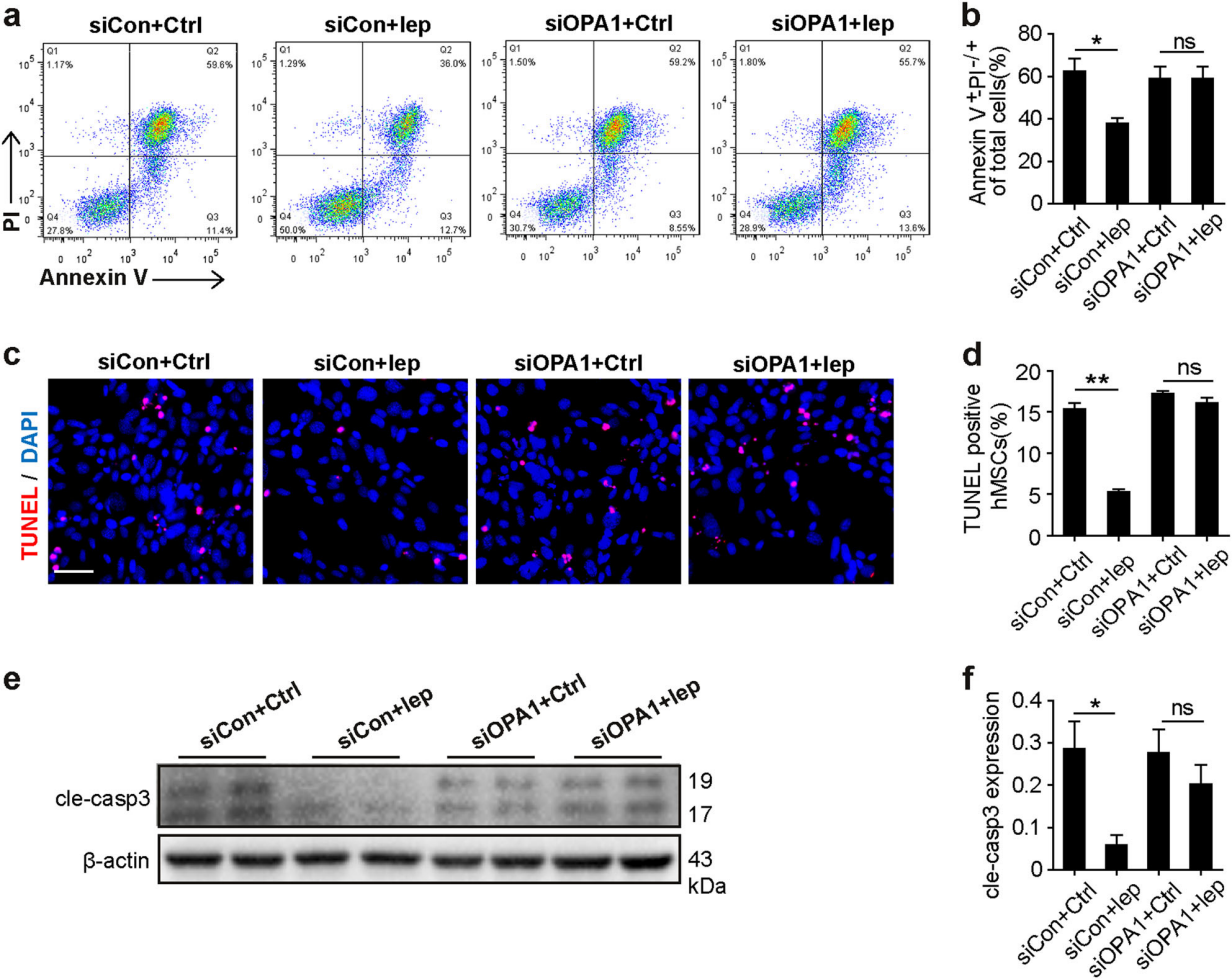
OPA1 was required for the protective effects of leptin in response to GSDH stress. **a**, **b** Annexin V/PI staining was performed to measure cellular apoptosis and necrosis, and early (Q3) and late (Q2) apoptosis events were quantified simultaneously. **c**, **d** Representative images of TUNEL staining and DAPI staining. Scale bar, 50 μm. Quantification of apoptotic cells by TUNEL-positive nuclei. **e**, **f** Cleaved caspase 3 (cle-caspase 3) protein expression of WCL was detected by western blot; β-actin served as a loading control. Independent experiments were performed three times. Data were shown as mean ± SEM. *denotes *P* < 0.05. **P < 0.01

Subsequently, we assessed whether OPA1 is indispensable for the leptin-mediated anti-apoptosis effects on hMSCs. Total OPA1 expression was knocked-down using siRNA specific for OPA1 (siOPA1), whereas scrambled siRNA (siCon) served as the control. The efficiency of the siOPA1 was confirmed by western blot (Supplementary Figure S6e). In the GSDH, we found similar results in siOPA1 groups that were treated with either leptin or control, as shown by both Annexin V/PI staining (Figs. 5a, b) and TUNEL staining (Figs. 5c, d). Also, the reduced levels of cleaved caspase 3 induced by OPA1 in hMSCs-Lep^pre^ were abolished by treatment with siOPA1 (Figs. 5e, f), suggesting that the leptin-induced cytoprotective roles are dependent on L-OPA1 accumulation.

### Leptin enhances OPA1 by inhibiting the activity of OMA1

To explore the underlying mechanisms, we assessed the transcriptional levels of OPA1 and found no significant difference (as shown above). Besides, previous evidence suggested redistributions of OPA1 into cytoplasm that was concomitant with that of cytochrome *c* release into the cytoplasm^31^, however, we failed to detect significant OPA1 release from mitochondria to the cytoplasm in hMSCs-Lep^pre^ and hMSCs-Ctrl^pre^ subjected to GSDH (data not shown).

**Fig. 6:**
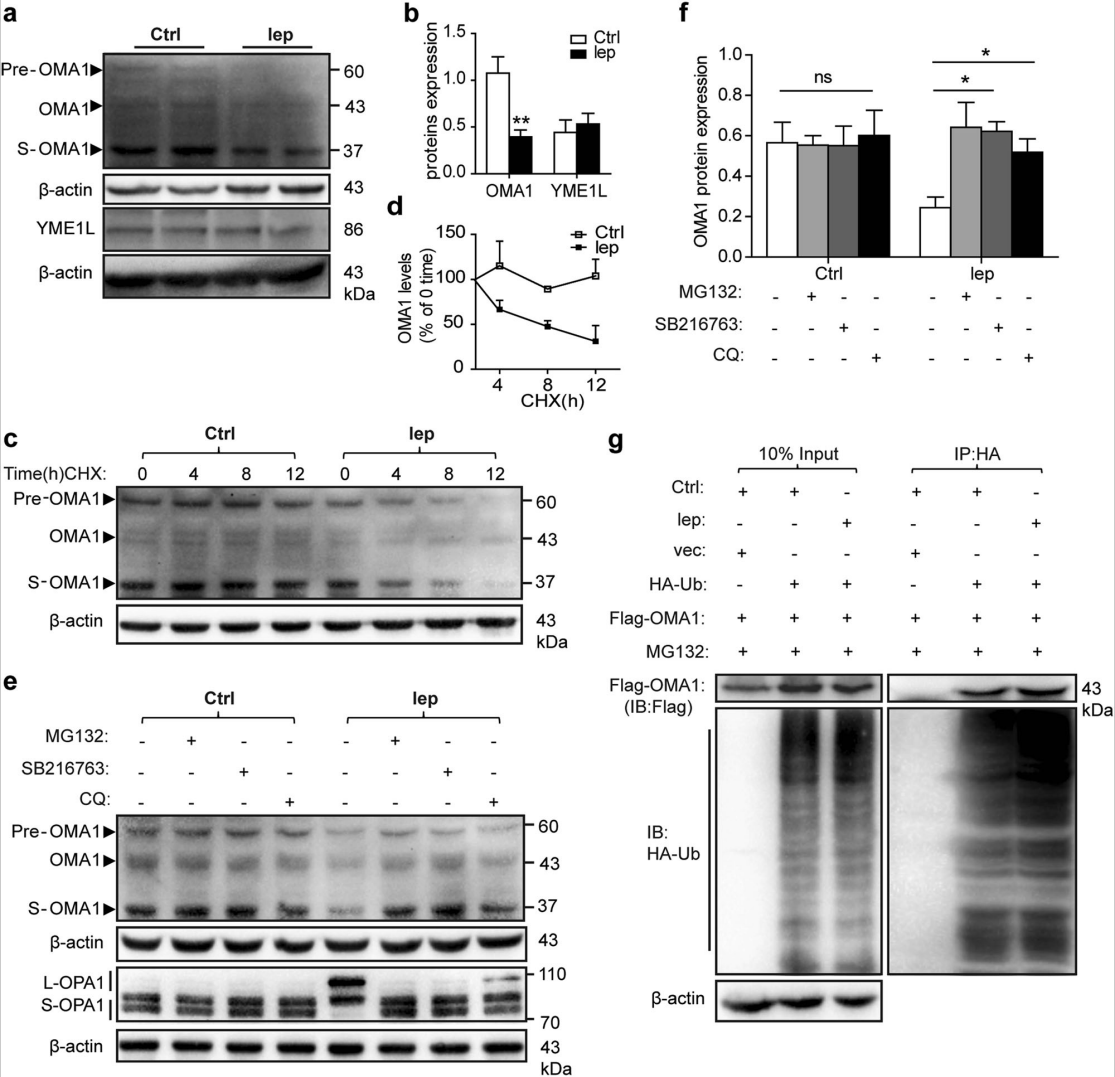
Leptin initiated OMA1 disruption through ubiquitin/proteasome-mediated mechanism. **a**, **b** OMA1 and YME1L were detected by western blot for both hMSCs-Lep^pre^ and hMSCs-Ctrl^pre^ cultured in GSDH for 24 h, and their protein levels were quantified by normalization to β-actin. **c**, **d** OMA1 protein level was quantified by western blot after hMSCs were treated with CHX (10 μg/ml) for the indicated duration using β-actin as a loading control, the OMA1 expression levels just before of CHX treatment were set as a baseline level. **e**, **f** Western blot of OMA1 and OPA1 isoforms protein levels of hMSCs-Lep^pre^ and hMSCs-Ctrl^pre^ were co-incubated with MG132 (2 μM), SB216763 (10 μM), or CQ (50 nM), respectively, in GSDH culture for 24 h and quantified in bar graphs. **g** HEK293T cells were transfected with Flag-OMA1 and HA-ubiquitin (HA-Ub) or empty vector for 48 h, and then, treated with MG132 (10 μM) for an additional 2 h; leptin (50 ng/ml) was administered throughout the whole period. WCLs harvested from these cells were explored by IP with anti-HA affinity gel; subsequently, Flag-OMA1 protein level was measured by western blot using anti-Flag antibody

Two mitochondrial inner membrane proteases, OMA1 and YME1L, are essential regulators of OPA1 processing. YME1L accelerates the mitochondrial fusion by cleaving OPA1 into L-OPA1, and OMA1 mediates mitochondrial fragmentation through transformation of S-OPA1^23,32^. Consequently, we tested whether YME1L and/or OMA1 were involved in leptin-mediated OPA1 upregulation. We found that leptin administration led to a reduction of OMA1 under GSDH (Figs. 6a, b). However, leptin did not affect OMA1 protein levels in normoxic-cultured cells (Supplementary Figures S7a and S7b), and no change was observed in the gene levels under either normoxic or GSDH culture conditions (Supplementary Figure S7c).

The activation of OMA1 depends on its cleaved form S-OMA1 (~34 kDa) rather than endogenous OMA1 (~40 kDa) as only the active form of S-OMA1 can cleave OPA1 to generate S-OPA1^33^. Thus, we analyzed OMA1 proteins by western blot. As compared with hMSCs-Ctrl^pre^, the expression of S-OMA1 was reduced in the hMSCs-Lep^pre^ (Figs. 6a, b), which accounted for the elevated L-OPA1 expression in hMSCs-Lep^pre^. Notably, neither protein nor gene expression level of YME1L was affected by leptin administration under either normoxic or GSDH conditions (Figs. 6a, b, Supplementary Figures S7a–S7c). Collectively, current data suggested that leptin promotes the mitochondrial fusion in an L-OPA1-dependent manner via inhibition of OMA1 activation without affecting YME1L.

### Leptin modulates instability of OMA1 through the ubiquitination-proteasome system

To investigate the mechanism underlying OMA1 downregulation, leptin-mediated hMSCs were administered cycloheximide (CHX), a general gene translation inhibitor at selective time points in GSDH. We found that CHX administration did not affect OMA1 expression, suggesting that leptin-mediated reduction of OMA1 might occur via the stability/degradation pathway (Figs. 6c, d). Furthermore, the ubiquitination-proteasome systems and autophagy–lysosome pathways are the two primary machineries for the degradation of proteins to maintain cellular homeostasis. This phenomenon was assayed by hMSCs that were treated with both leptin and the 26 S proteasome inhibitor, MG132. Nevertheless, we found that the degradation of OMA1 was interfered by MG132. Interestingly, only a slight reduction of OMA1 was observed by chloroquine (CQ), an autophagy inhibitor (Figs. 6e, f). Taken together, ubiquitination is mainly responsible for the degradation of OMA1 induced by leptin treatment. Importantly, these data indicate that L-OPA1 isoforms are negatively correlated with OMA1 expression, and these observations were consistent with previous studies that OMA1 cleaved OPA1 to transform into S-OPA1^33^.

To further investigate the ubiquitination of OMA1, we transfected the HEK293T cells with HA-tagged Ubiquitin plasmid (HA-Ub) and Flag-OMA1 expression plasmids. Subsequently, the cells were treated with MG132 before collection for analysis. Flag-OMA1 could be pulled down in HEK293T cells transfected with both HA-Ub and Flag-OMA1 plasmids, but not from the cells transfected with an only Flag-OMA1 plasmid (Fig. 6g), designated OMA1 as an ubiquitinated protein. In addition, the elevated ubiquitination levels of Flag-OMA1 in the leptin-treated group (Fig. 6g), suggesting leptin promoted OMA1 ubiquitination.

### Ubiquitination of OMA1 is dependent on phosphorylation of GSK3

**Fig. 7:**
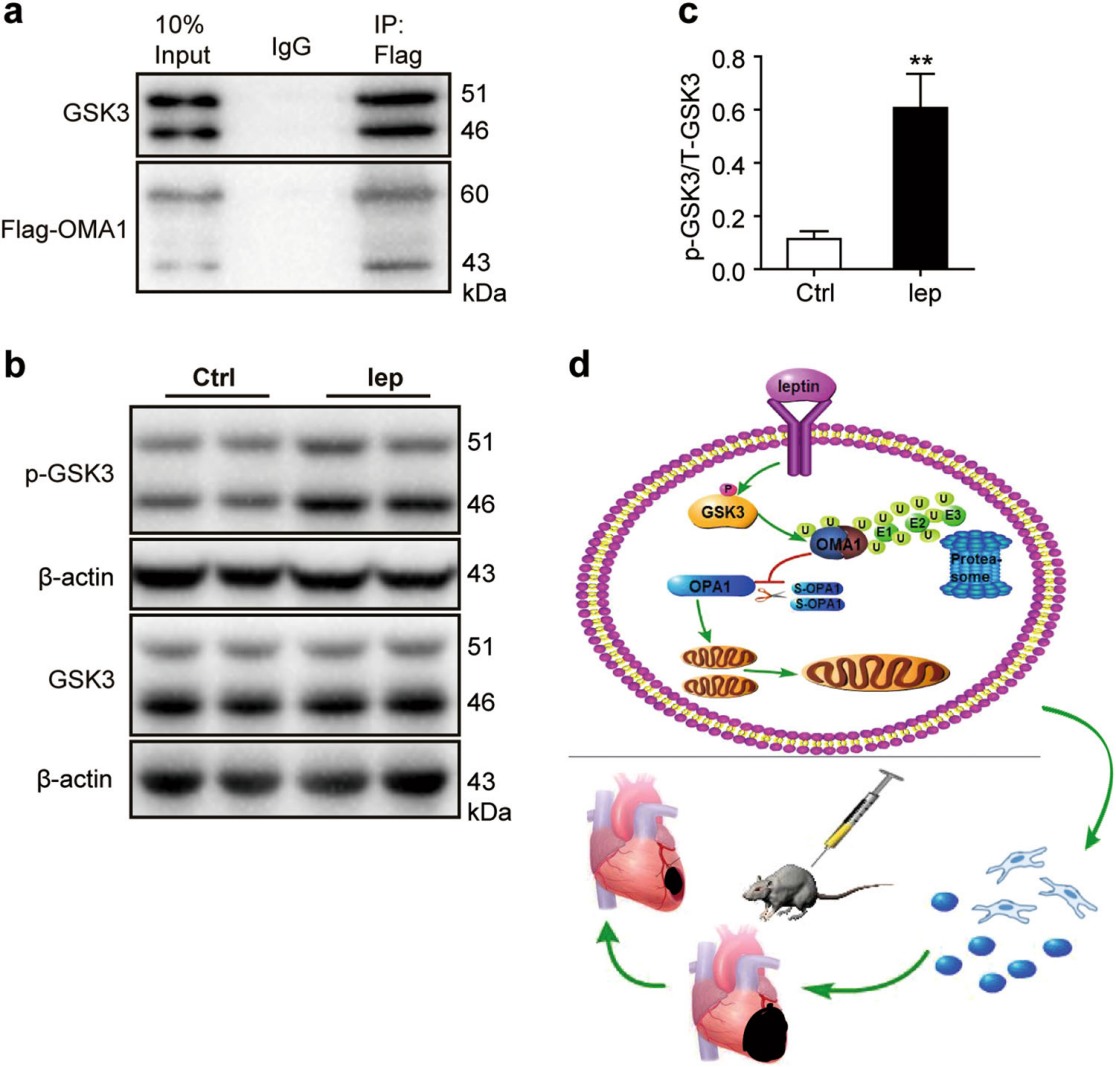
Leptin-accelerated OMA1 destruction depends on phosphorylation of GSK3. **a** HEK293T cells transfected with Flag-OMA1 plasmid was pulled down via co-IP analysis with monoclonal anti-flag M2 antibody. GSK3 protein expression was detected by western blot using anti-GSK3 antibody. **b**, **c** Phosphorylation of GSK3 (Tyr-216/Tyr-279) was detected by western blot and protein bands from hMSCs-Lep^pre^ and hMSCs-Ctrl^pre^ quantified by densitometry. **d** A schematic illustration showed that leptin protected mitochondrial integrity through the OPA1-dependent pathway, which was due to OMA1 ubiquitination to enhance hMSCs survival and increased the paracrine of hMSCs rescued heart function

During the ubiquitination process, before recognition by ubiquitin, the substrates usually undergo a phosphorylated modification within the target motifs; for example, Cdc4 phosphodegron that is usually activated by GSK3^34,35^. In addition, GSK3 can mediate the proteolysis of OMA1 during the first and second mitosis in *C. elegans*^36^. Consecutively, leptin mediates the GSK3 activity to enhance glucose uptake^37^. Therefore, we speculated that leptin modulates the OMA1 degradation via GSK3-dependent OMA1 ubiquitination under GSDH. Flag-OMA1 plasmid was transfected into HEK293T cells, and the interaction between Flag-OMA1 and endogenous GSK3 was evaluated by co-immunoprecipitation (co-IP), which revealed that GSK3 was pulled down in the case of Flag-OMA1 but not IgG control only cells (Fig. 7a), thereby suggesting a reciprocal relationship between OMA1 and GSK3.

To investigate the role of GSK3 in leptin-mediated OMA1 degradation, hMSCs were treated with leptin in the presence or absence of GSK3 inhibitor (SB216763). Markedly, SB216763 blocked the degradation process of OMA1, increasing S-OPA1 expression (Figs. 6e, f).

Moreover, GSK3 phosphorylation at different sites generates diverse results, including phosphorylation of Tyr-216/Tyr-279 that is associated with enhanced GSK3 activity, whereas phosphorylation of Ser-9/Ser-21 participates in the inactivation of GSK3^37^. The present study detected a high phosphorylation of GSK3 at Tyr-216/Tyr-279 residues in hMSCs-Lep^pre^ (Figs. 7b, c), implicating leptin-induced GSK3 activation via phosphorylation of Tyr-216/Tyr-279 sites. Taken together, the current data was in agreement with the pathway wherein leptin-induced phosphorylation of GSK3 at Tyr-216/Tyr-279-facilitated OMA1 degradation by the ubiquitination/proteasome machinery to elevate L-OPA1 expression. This in turn, enhanced the mitochondrial fusion and integrity, which improve the survival of hMSCs (Fig. 7d).

## Discussion

In this study, we detected that implanted hMSC_lep_ infected by lentivirus overexpressing leptin in MI-mice heart had an improved survival rate and less-cardiac dysfunction at day 28 post MI in vivo, which was contributed by the paracrine activity of hMSCs. Similarly, we observed an enhanced and elongated tubular mitochondrial networks in hMSCs-Lep^pre^ subjected to ischemia-like conditions in vitro, suggesting that mitochondrial integrity maintenance has a vital role in the apoptotic resistance. Furthermore, we verified that leptin conferred the protection of hMSCs against anti-apoptosis through enhanced mitochondrial fusion and integrity. Although impaired fusion correlates with disease, the hyperfused mitochondrial networks can resist apoptosis, which was constant with stress-induced mitochondrial hyperfusion^38^. Also, the administration of leptin did not increase cell proliferation under the basal state (Supplementary Figure S7d). Mechanistically, we found that the apoptotic resistance of hMSCs-Lep^pre^ was dependent on OPA1, which maintained the mitochondrial integrity and inhibited the release of pro-apoptotic proteins^22,31,39^. Next, the ubiquitination-dependent degradation of OMA1 was deduced to play a fundamental role in the enhancement of OPA1. In addition, the phosphorylation of GSK3 appears to directly regulate OMA1 ubiquitination. The current data provided a pharmacological approach to potentiate stem cell therapeutic efficacy of cardiovascular indications.

Increasing studies showed that the therapeutic effects of MSCs were primarily contributed by paracrine function^25–27^. In addition, we found that hypoxia-preconditioned MSCs enhanced angiogenesis in MI-nonhuman primate model previously^28^. We further verified that leptin signaling in hMSCs is a potential paracrine mediated to improve angiogenesis, which was in agreement with a previous report stating that higher concentrations of leptin were observed in the conditioned medium of MSCs^25^. Consisted with previous reports^30,40^, leptin promoted the angiogenesis of surviving hMSCs and we found that leptin-induced tube formation was similar to the effect of hMSCs-conditioned medium.

Most common related concern of MSCs injection is the MSCs-induced inflammation or immuno-reaction. Increased inflammation has been induced when MSCs differentiated into adipocytes^41^. However, it has been generally shown that MSCs have the ability to have immunomodulation effects via regulating CD4 T and CD8 T cells^1,42,43^. Our previous studies report that the immune response of MSCs transplantation has no difference compared with DMEM group in nonhuman primate model^28^. Being consistent with these previous studies, our current data demonstrated that quantification of immunoflourescence staining of CD3, CD8 and CD68 had no significant difference among DMEM, hMSC_vec_, and hMSC_lep_ groups (Supplementary Figures S8a and S8b).

Moreover, some detrimental effects of leptin are reported. Leptin activates matrix metalloproteinase-2 that is involved in pathological myocardial matrix remodeling either in neonatal rat cardiac myofibroblasts^44^ or in cultured human ventricular cardiomyocytes^45^. Leptin provokes vascular thromdel of carotid arterial^46,47^. Interestingly, however, leptin-mediated protection against apoptosis via decreased DNA damage has been reported^48^. A switch towards glucose metabolism induced by leptin attenuates the morbidity in an MI-mice model, associated with STAT3-PI3K-Akt signaling^49^. Moreover, direct cardioprotective effects of leptin have been verified in other studies^50–52^. The current results revealed the influence of hMSC_lep_ on heart function but not the directly injection of leptin. Of cause, other group and our group have previously examined that a few of resident MSCs at 14 days post MI^26,28^. Therefore, we assumed that leptin could not be generated from hMSC_lep_. It is hardly believed that leptin would make the detrimental effects on heart for a long-term of post MI. Nevertheless, the therapeutic effects of MSCs are primarily contributed by paracrine, which is consistent with several previous studies^25,27,29^.

Intriguingly, increased ATP production was found in hMSCs-Lep^pre^ post GSDH injury. For energy production, hMSCs metabolism is mainly dependent on glycolysis even under normoxia with exceptions that hMSCs rely on mitochondria after differentiation^53,54^. In consistency with earlier findings, we found elevated production of lactate in supernatants and less in cells (Supplementary Figure S7e), which indicates that surviving hMSCs relied primarily on glycolytic ATP production after leptin administration. The current results are in agreement with a recent report stating that reduced cellular lactate facilitates MSCs survival related to MCT4 upregulation^26^. Taken together, despite insufficient mitochondrial respiration, we speculated that leptin-modulated mitochondria act as a harbor for cristae maintenance and resistance to release of pro-apoptotic factors but not enhanced energy generation.

As a dynamin-like GTPase inner membrane protein, OPA1 is a crucial regulator to balance the IMM fusion and fission for stabilizing cristae structure and reducing the cytochrome *c* release to resist apoptosis^12,22,39^. OPA1 overexpression protects the mouse heart from ischemia and sliver apoptosis^39^. OPA1 knockout mouse embryonic fibroblasts exhibit increased cell death^55^. Loss of OPA1 leads to mitochondrial fragmentation^22^. OPA1 is composed of at least eight isoforms, in the form of L-OPA1 and S-OPA1. Interestingly, different isoforms of OPA1 cause diverse results. L-OPA1 is correlated with fusion, whereas S-OPA1 leads to fission^12,24^. We observed that leptin treatment caused elevated protein levels of L-OPA1 but no change in the mRNA levels.

Interestingly, two mitochondrial inner membrane proteases, OMA1 and YME1L, are responsible for OPA1 proteolytic processing to balance IMM dynamic^23,32^. The two proteins are target-specific sites under different stimuli. YME1L cleaves OPA1 at S2 and S3 site to transform into L-OPA1 to induce fusion when cells are faced with increased oxidative phosphorylation, whereas OMA1 cleaves OPA1 at an S1 site to transform into S-OPA1, resulting in the fragmented response to cellular stress, mitochondrial dysfunction, or deletion of YME1L^12,23^. We discovered that OMA1 activation was attenuated in hMSCs-Lep^pre^ response to the GSDH condition independent on YME1L. In addition, membrane depolarization of hMSCs-Lep^pre^ and hMSCs-Ctrl^pre^ were detected under GSDH, and these results were in agreement with previous reports that membrane depolarization and the presence of YME1L are two essential elements for stress-induced OMA1 activation by self-cleavage^12,32,33^. Besides, increased glycolysis observed in the hMSCs-Lep^pre^ group might be associated with OMA1 activation as a previous study reported that OMA1 activation induced a metabolic switch from oxidative to glycolytic metabolism in cardiomyocytes^12^. As a metalloprotease, OMA1 primarily functions as a mitochondrial proteolytic activity for misfolded or damaged proteins, and calpain 3 is the domain harbored in the molecule^24^. Interestingly, we further observed OMA1 degradation after leptin-mediation, which was also consistent with an earlier report of an ATP-dependent step in OMA1 degradation^32^. Collectively, we can conclude that leptin promotes OMA1 destruction that blunts OPA1 proteolysis in the cells and leads to hyperfusion as well as suppression of the downstream trigger points.

In terms of proteins degradation, the current data demonstrated that leptin-mediated degradation of OMA1 is controlled primarily by the ubiquitination-proteasome system with minimal input by autophagy, also consistent with the previous finding that OMA1 is recognized by the 26 S proteasome^56^. In embryonic development of *Caenorhabditis* *elegans*, GSK3 is required to mediate the proteolysis of OMA1^36,57^. Similarly, GSK3 activation is required for the degradation of multiple other substrates (such as c-Myc, β-catenin, and p100) via recruited E3 ubiquitin ligase^35,58,59^. Herein, we ascertained a direct interaction relationship between GSK3 and OMA1, and ubiquitination of OMA1 was regulated by GSK3 phosphorylation. Significantly, we verified leptin-mediated activation of GSK3 through phosphorylation of Tyr-216/Tyr-279 sites but not Ser-9/Ser-21 (phosphorylation of Ser-9/Ser-21 indicates GSK3 inactivation)^37^. Specifically, leptin-primed GSK3 activation triggered OMA1 for ubiquitination and degradation, thereby facilitating L-OPA1 accumulation independent of YME1L.

## Conclusion

The present study, for the first time, demonstrated a crucial role of mitochondrial integrity in leptin-mediated anti-apoptosis of engrafted hMSCs in ischemic environments. Mechanically, cardioprotection of leptin was dependent on OPA1 and maintenance of mitochondrial cristae architecture. Furthermore, leptin-induced ubiquitination of OMA1 was reported via a GSK3-dependent pathway. These results may provide novel pharmacological avenues to potentiate the therapeutic efficacy of hMSCs therapy for cardiovascular indications.

## Materials and Methods

### Animals model of myocardium infarct and hMSCs treatment

All procedures were conducted in accordance with the guidelines of the National Health and Family Planning Commission of the People’s Republic of China and approved by the Animal Use Committee of Zhejiang University. MI model was performed on 10–12-week-old male C57BL/6 J mice (Zhejiang Chinese Medical University), 20–25 g, by ligation of the left anterior descending coronary artery (LAD) as reported previously^7^. Immediately after the ligation of LAD, hMSC_vec_, or hMSC_lep_ (both 1.5 × 10^5^ cells per mouse) suspended in 20 µL DMEM (Corning, Manassas, VA, USA) were implanted to the border zone of the infarct heart via direct injection into four sites, whereas equivalent volume DMEM was injected into the control mice.

### C**ells isolation, culture, and glucose/serum-deprived/hypoxia injury**

Human bone marrow-derived MSCs were isolated from patients operated for hip replacement, as reported previously^7^, and used between passages 4–8. Informed consents were obtained from donors, and the protocol was approved by the Human Ethics Committee of the Second Affiliated Hospital of Zhejiang University. Cells were cultured in DMEM with low glucose (LG) with 10% (v/v) fetal bovine serum (FBS; Life Technologies, Paisley, UK) and 100 U/ml penicillin/streptomycin (v/v) (SP) at a density of 1 × 10^4^ cells/cm^2^ in a humidified atmosphere with 95% air / 5% CO_2_ at 37 °C.

At 24 h after plating, hMSCs were pretreated with human recombinant leptin (50 ng/ml, R&D Systems, Minnesota, USA) in LG DMEM containing 1% (v/v) FBS under normoxia cultured condition for 24 h, while the solvent for leptin was used as control group. After replacement of DMEM without glucose and FBS, hMSCs were also cultured with leptin (50 ng/ml) under hypoxia condition (0.5% O_2_/5% CO_2_) at 37 °C for an additional 24 h. The concentration of leptin was selected based on other studies in vitro^16,60^.

### Cell siRNA transfection

siRNA targeting human OPA1 (siOPA1) and control siRNA (siCon) were purchased from GenePharma (Shanghai, China). siRNA transfection was conducted using Lipofectamine RNAi MAX (Invitrogen, Carlsbad, CA, USA) according to the manufacturer’s instructions. hMSCs were transfected with three short siRNAs (50 nM) targeting different specific sequences of OPA1 (online Supplementary Table S3). We selected siOPA1-#2, which resulted in a drastic knockdown, for use in subsequent studies. After 48 h transfection, hMSCs were further pretreated with leptin or control for an additional 24 h, followed by exposure to GSDH condition.

### Plasmid vectors and transfection

Both full-length Flag-tagged human OMA1 plasmid (Flag-OMA1) (GeneChem) and HA-Ub (Miaoling Bioscience, Wuhan, China) were cloned into vectors containing the CMV promoter. Plasmid transient transfections were performed using Lipofectamine 3000 transfection reagent (Life Technology, Waltham, MA, USA) according to the manufacturer’s protocol.

### Recombinant lentivirus vector construction and cell infection

Recombinant lentivirus expressing full-length human leptin tagged with flag (Flag-lep) was provided by GeneChem. Lentivirus containing empty vector served as controls. hMSCs were transduced by lentiviral vectors as reported previously^18^. In brief, hMSCs were mixed with purified viral vectors at multiplicities of infection of 50 (for lentivirus) and 20 (for polybrene at a final concentration of 8 μg/ml, Sigma) overnight. After replacement of viral suspension to fresh medium, cells were cultured for another 24 h. The quantification of infection was analyzed by western blot analysis of the target gene, and fluorescent microscopy identified the GFP-positive cells.

At 48 h after infection of lentivirus, the hMSCs were harvested to be used for the cells engraftment in vivo. Also, some of the infected hMSCs were exposed to hypoxia condition (0.5% O_2_/5% CO_2_, at 37 °C) in DMEM without glucose and FBS for an additional 24 h in vitro.

### Sirius Red staining

Mice were killed with sodium pentobarbital (50 mg/kg intraperitoneally) and hearts were harvested and embedded in paraffin. Tissue samples were cut into 3-μm thick sections, and were stained with Sirius Red (Solaribio, Beijing, China) for infarct zone evaluation as described previously^61^. Infarct areas were calculated by the sum of the endocardial and epicardial length of the infarct zone in proportion to the total length of the endocardial and epicardial left ventricle using Image-Pro-Plus software (Media Cybernetics, Rockville, MD, USA).

### Immunostaining

Hearts were embedded in Tissue Tek O.C.T. compound (Sakura Finetek, Torrance, CA, USA) and cut into 7-μm thick sections. Tissues slices were fixed with 4% paraformaldehyde and permeabilized with 0.2% Triton for 10 min each, followed by blocking using 3% bovine serum albumin (BSA). Subsequently, samples were incubated with primary antibody overnight at 4 °C, followed by incubation of conjugated secondary antibody for 1 h at room temperature. Immunofluorescence images were observed with × 400 objective, and images captured by confocal microscopy (Leica, Wetzlar, Germany). The antibodies are listed in the supplemental information.

### TUNEL staining

Tissue or cell samples were fixed with 4% paraformaldehyde and permeabilized with 0.2% Triton as described above, and then, incubated with TUNEL (Roche Applied Science, Indianapolis, IN, USA) reaction compound for 1 h at 37 °C in the dark according to the manufacturer’s instructions. Nuclei were stained with dihydrochloride (Vector, Burlingame, CA, USA). For heart tissue, the apoptotic ratio was calculated as the percentage of cells (nuclei counting) that were positive for both TUNEL and GFP in total GFP-positive cells. In the case of cell samples, the apoptotic ratio was calculated as TUNEL-positive cells in total cells/nuclei. The images were obtained from 8 to 10 randomly selected fields in each sample or per well.

### Annexin V/PI staining

After washing with phosphate-buffered saline, the harvested cells (2 × 10^5^ cells per sample) were incubated with a mixture of Annexin V and PI dye for 30 min according to the manufacturer’s instruction of Annexin V/PI Apoptosis Detection Kit (Dojindo, Minato-ku, Tokyo, Japan) and quantified by flow cytometry (BD Biosciences, San Jose, CA, USA).

### OCR

Oxygen consumption was measured in the intact cells as described previously^18^. In brief, the cultured cells were harvested and resuspended with MiR05 (containing 0.5 mM EGTA, 3 mM MgCl_2_•6H_2_O, 60 mM potassium lactobionate, 20 mM taurine, 10 mM KH_2_PO_4_, 20 mM HEPES, 110 mM sucrose, and 1 g/l fatty acid-free BSA at pH 7.1). Subsequently, the OCR was measured by using OROBOROS Oxygraph-2k at 30 °C (Oroboros Instruments, Austria). Then, different mitochondrial enzymes of mitochondrial respiratory chain complexes were used to record the OCR: glutamate (5 mM)/malate (2.5 mM) for complex I, succinate (10 mM)/rotenone (1.25 mM) for complex II, and TMPD (0.5 mM)/ascorbate (2 mM)/antimycin A (5 µM) for complex IV.

### Flow cytometry for cell characterization and ψMt

Passages 4–8 of hMSCs were characterized as described previously^62^. In brief, hMSCs were harvested and incubated with the surface molecular specific antibodies (mesenchymal surface markers: allophycocyanin-CD90, PE-CD29, and PE-CD105; endothelial cell surface marker: FITC-CD34, PE-CD31, hematopoietic surface marker: FITC-CD117 and isotype-matched control) for 1 h at room temperature, and the expression of cell surface markers analyzed using flow cytometry.

Moreover, hMSCs were incubated with TMRM (50 nM) for 30 min at 37 °C, and then, harvested for evaluating the ψMt using flow cytometry with a BD FACSCount II Flow Cytometer (BD Biosciences, San Jose, CA, USA).

### Cell proliferation assay

hMSCs were detected by Cell Counting Kit 8 (CCK-8) as our previous protocol^62^. In brief, after treatment with leptin at different concentrations of 50 and 100 ng/ml at 24, 48, and 72 h, the mixture of 100 μl medium with 10 μl CCK-8 (Dojindo, Minato-ku, Tokyo, Japan) was added into 96-well plates (3000 cells/sample). After incubation for 2.5 h at 37 °C, the absorbance was measured at 450 nm by a microplate reader (Bio-Rad, Berkeley, CA, USA).

### H_2_O_2_-induced apoptosis assay

After leptin (50 ng/ml) pretreatment for 24 h, hMSCs were subjected to H_2_O_2_ (500 μM) with leptin (50 ng/ml) or solvent control in DMEM without glucose and FBS (95% air/5% CO_2_, at 37 °C) for 4 h in vitro.

### Tube formation of HUVECs co-cultured with leptin alone or supernatant of hMSCs pretreated with leptin

hMSCs (1 × 10^6^ cells) were pretreated with leptin (50 ng/ml) or control in DMEM without FBS under normoxic culture for 24 h, following which, the conditioned media were collected. In addition, the conditioned media obtained from DMEM with leptin (50 ng/ml) or control-alone, but not hMSCs or FBS, for 24 h were also collected. HUVECs were plated at 5 × 10^4^ cells/well in the above different conditioned media in 24-well plates that were precoated with Matrigel (BD, Franklin Lakes, New Jersey, USA). After 4 h of plantation, the tube formation of HUVECs was quantified by counting the total number of interbranch in each well using the Image-Pro software.

### Immunoprecipitation and western blot analysis

After transfected with 3 μg Flag-OMA1 plasmid for 48 h, HEK293T cells (5 × 10^6^ cells) were harvested in immunoprecipitation (IP) lysis buffer (containing 50 mM Tris-HCl-pH 7.4, 150 mM NaCl, 0.1% NP-40 and protease inhibitors). In total, 10% cell lysates were extracted for input, and the remaining supernatant was incubated in IP washing buffer containing monoclonal anti-flag M2 antibody (Sigma-Aldrich, Saint Louis, MO, USA) or normal mouse IgG (Santa Cruz Biotechnology, CliniSciences, Nanterre, France) at 4 °C overnight by gentle agitation. On the following day, the protein-antibody immunocomplexes were precipitated using precleared protein A/G beads (Santa Cruz Biotechnology) for an additional 2 h at 4 °C. Agarose with protein-antibody immunocomplexes was washed, harvested, and analyzed via immunoblotting with anti-GSK3 and Flag antibodies.

Western blot was performed as described previously^7^. In brief, the cells were lysed with RIPA buffer on ice, clarified by centrifugation and equivalent amounts proteins were used. The antibodies are listed in the supplemental information.

### Protein stability assay

hMSCs were treated with CHX (10 μg/ml) (Selleck Chemicals, Houston, Texas, USA) at the indicated time points under GSDH and harvested for the preparation of whole-cell protein lysates for western blot.

### Ubiquitination assay

hMSCs were co-incubated with leptin plus MG132 (2 μM) (Selleck Chemicals), SB216763 (10 μM) (Jinpu Biotechnology, Wuxi, China) or CQ (50 nM) (Selleck Chemicals), respectively, for 24 h in GSDH; cellular protein was harvested for western blot.

In addition, HEK293T cells (5 × 10^6^ cells) were transfected with 1.5 μg of each plasmid encoding Flag-OMA1 and/or HA-Ubiquitin (HA-Ub) and then cultured in DMEM with high glucose without FBS and SP medium for 24 h post transfection, the media were replaced and cells incubated at an additional 24 h. HEK293T cells were treated with MG132 (10 μM) for 2 h before harvesting for IP. HA-Ub proteins were pulled down using Red anti-HA affinity gel (Sigma-Aldrich, Missouri, USA) overnight and HA-Ub protein collected on the following day. The results were revealed by western blot using Flag-tag and HA-tag antibodies.

### Quantitative real-time PCR

Total RNA was extracted using Trizol reagent (Invitrogen, ThermoFisher Scientific) according to the manufacturer’s instructions. The gene expression levels were examined by real-time PCR using SYBR Green PCR Master Mix (Takara, Japan) and detected with NanoDrop spectrophotometer (Thermo). The expression data relative to β-actin were presented using the 2^-ΔΔCT^ method. The gene primers are shown in Supplementary Table S2.

### TEM

TEM was used to detect the mitochondrial network ultrastructure of hMSCs. In brief, the specimens were fixed with 2.5% glutaraldehyde for > 4 h. After washing three times with phosphate-buffered saline, the specimens were post-fixed with 1% OsO_4_ for 1–2 h. Next, the specimens were dehydrated by an ethanol gradient, followed by acetone for overnight infiltration. Furthermore, the specimens were embedded in Spurr resin and sectioned in Leica EM UC7 Manufacturer (Leica, Wetzlar, Germany). The sections were stained with uranyl acetate and alkaline lead citrate, and the image procured by Hitachi Model H-7650 TEM. The images were obtained randomly to measure the mitochondrial length using Photoshop software at × 30,000 magnification.

### Echocardiography

Echocardiography was performed at day 0, 3, 28 post MI surgery as described previously^62^. Mice were anesthetized by isoflurane inhalation. Two-dimensional and M-mode images were obtained and analyzed cardiac morphology and function with a Vevo 2100 system (VisualSonics, Toronto, Ontario, Canada). LVESD and LVEDD were measured for at least three interval cardiac cycles.

### Statistical analysis

Data with normal distribution were presented as mean ± SEM. Student’s *t*-test was used to determine significance in comparison of two sets of data. One-way analysis of variance followed by Tukey’s post test was used for comparison of more than three sets of data. All experiments were repeated at least three times. All data were analyzed using SPSS (version 17.0) statistical software. The statistics were presented by GraphPad Prism 5. *P* < 0.05 was considered as statistically significant.

## Electronic supplementary material

Figure S1

Figure S2

Figure S3

Figure S4

Figure S5

Figure S6

Figure S7

Figure S8

Table S1

Table S2

Table S3

Supplemental information

Supplementary figure legends
