# Supplementary figures and images for: Leptin increases mitochondrial OPA1 via GSK3-mediated OMA1 ubiquitination to enhance therapeutic effects of mesenchymal stem cell transplantation
Source: Cell Death Dis. 2018 May 10;9(5):556. doi: 10.1038/s41419-018-0579-9 (PMC5945599; doi:10.1038/s41419-018-0579-9)

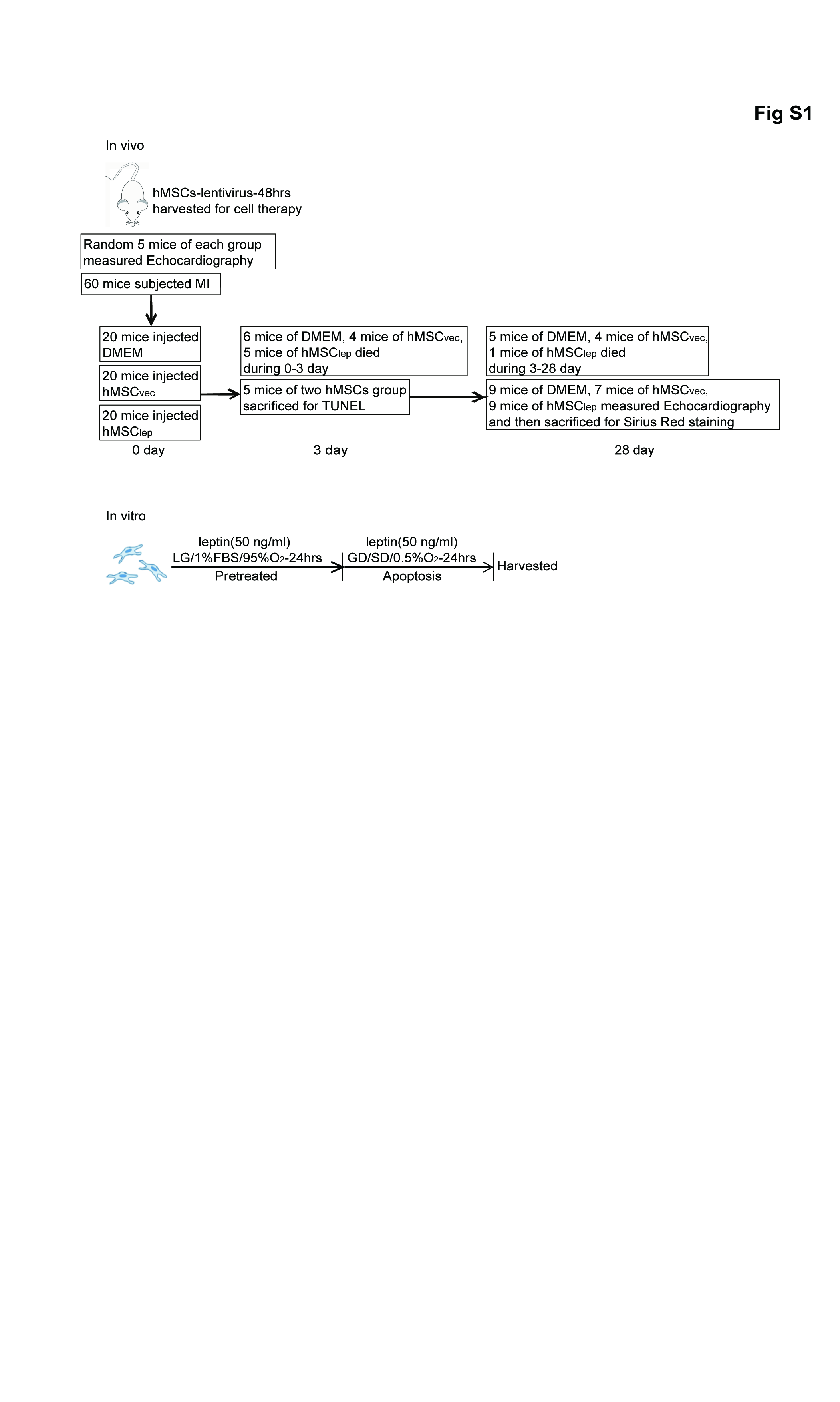

Supplement: Supplementary file 1 — Figure S1 [file 41419_2018_579_MOESM1_ESM.tif]

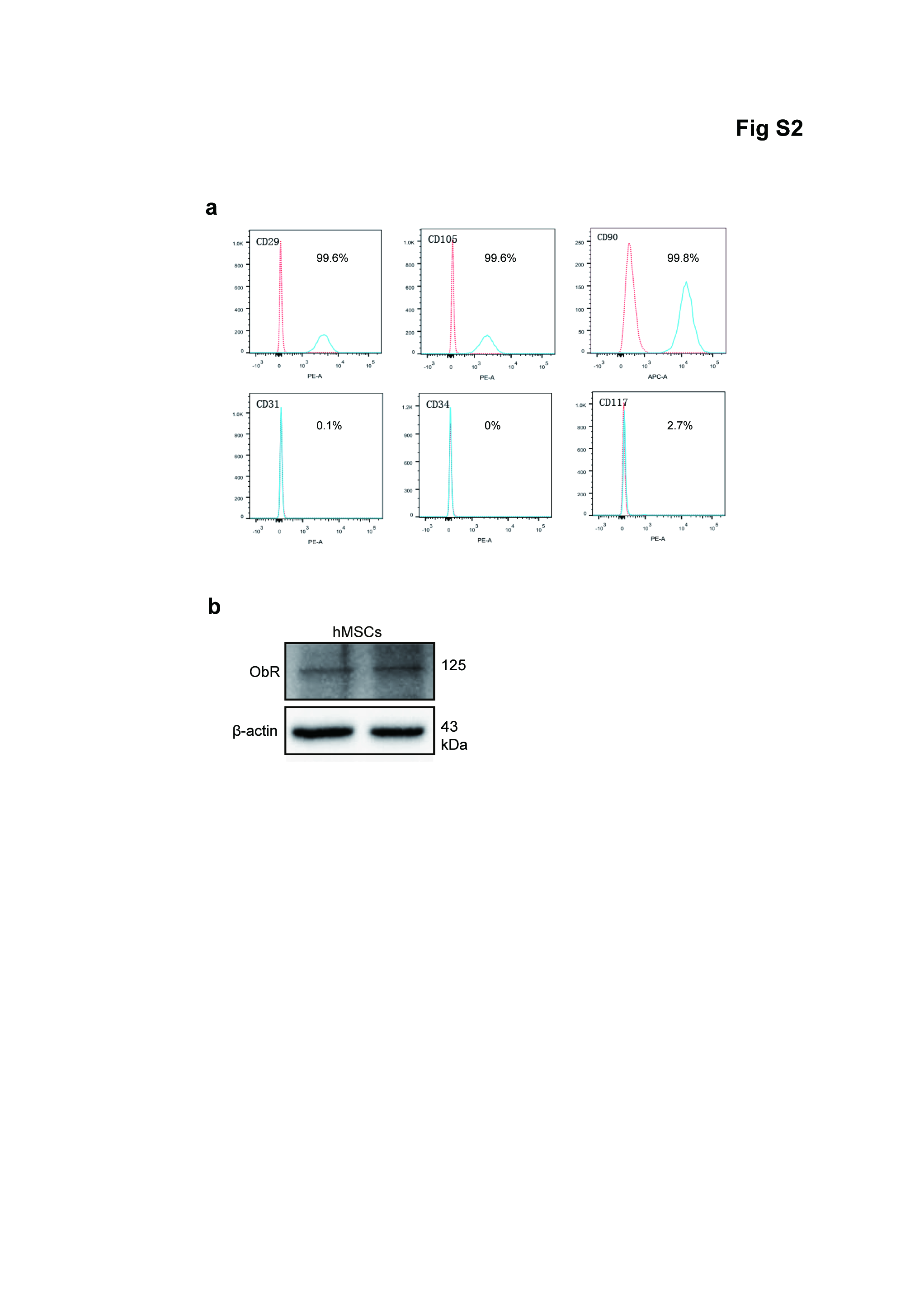

Supplement: Supplementary file 2 — Figure S2 [file 41419_2018_579_MOESM2_ESM.tif]

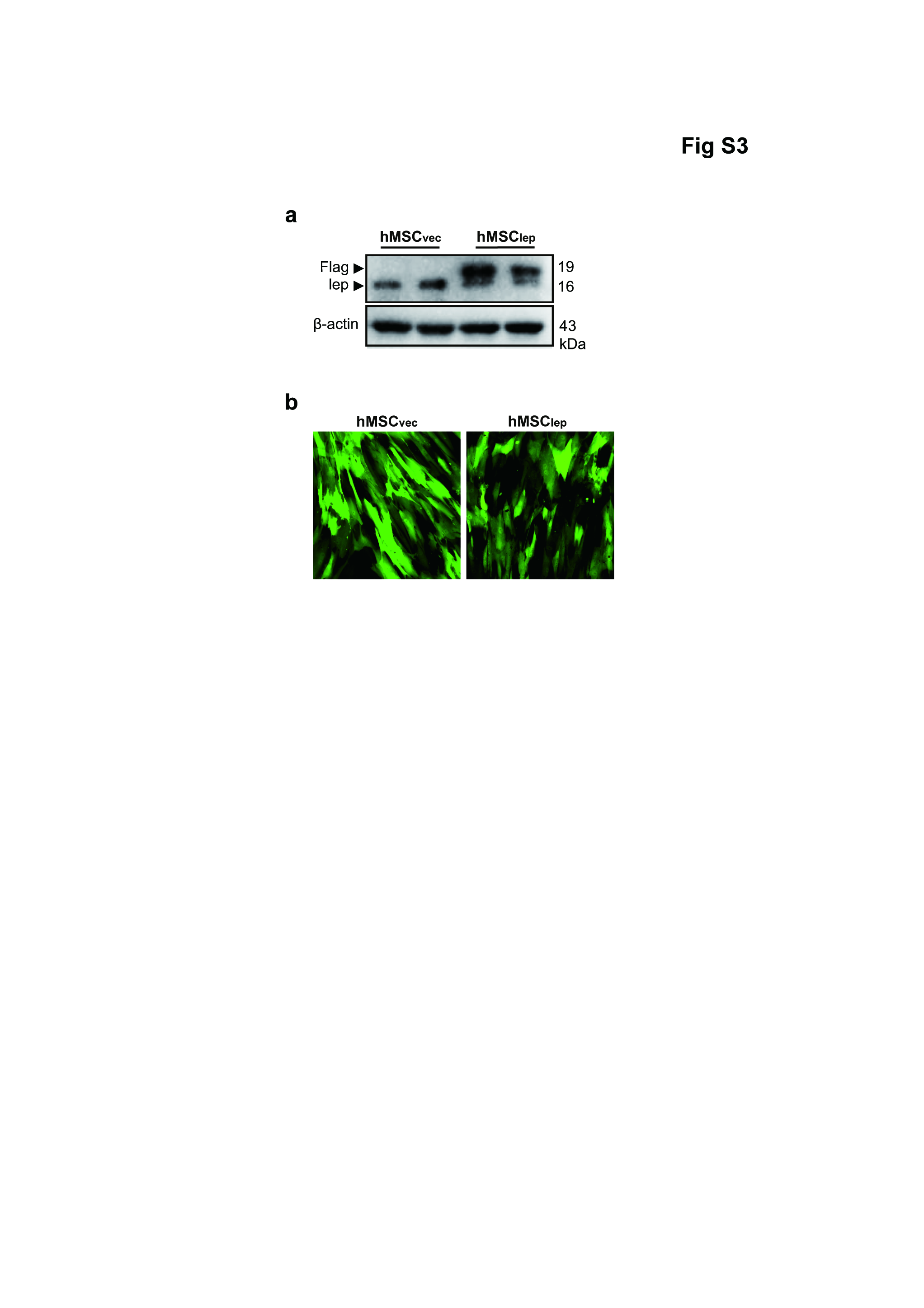

Supplement: Supplementary file 3 — Figure S3 [file 41419_2018_579_MOESM3_ESM.tif]

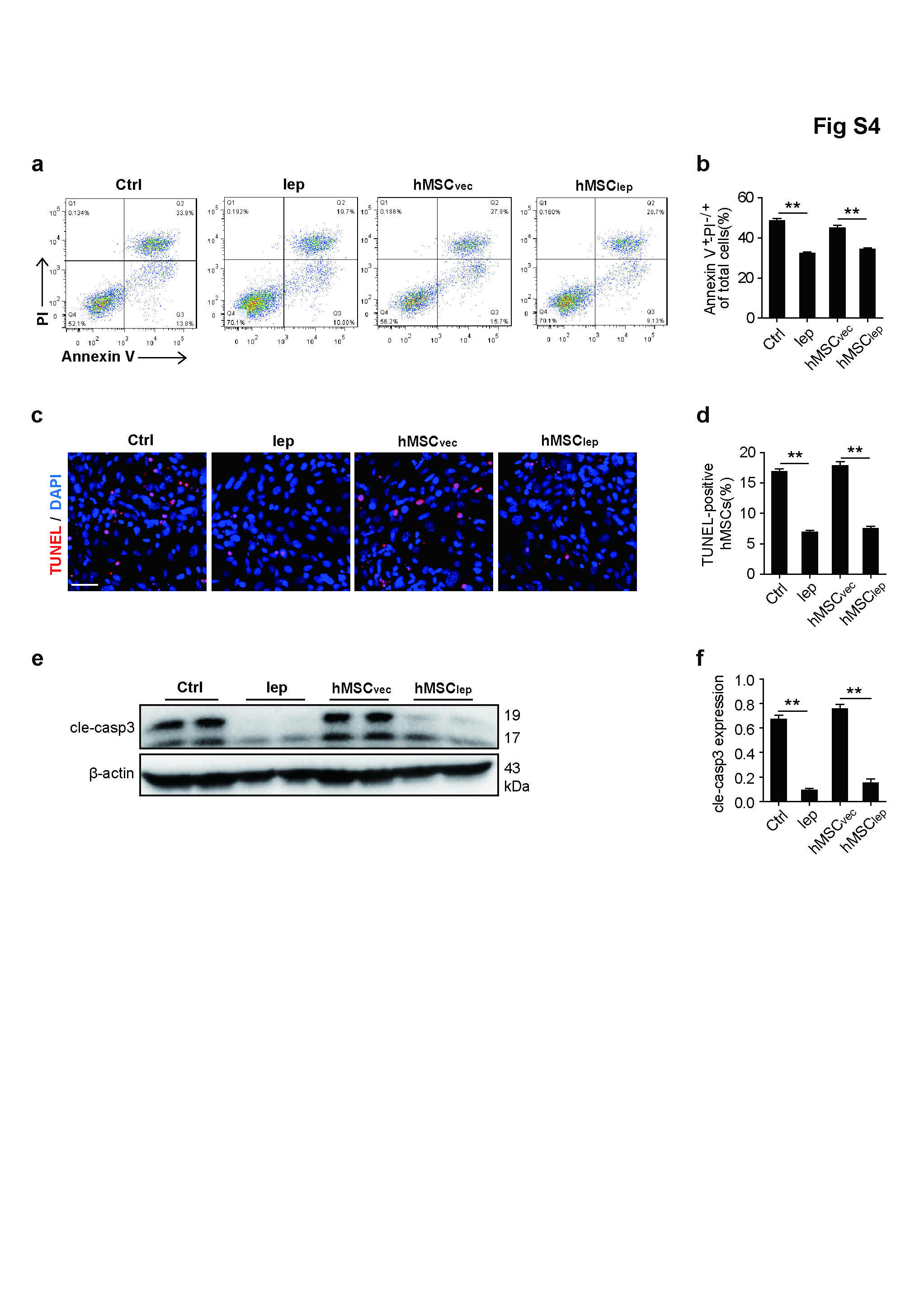

Supplement: Supplementary file 4 — Figure S4 [file 41419_2018_579_MOESM4_ESM.tif]

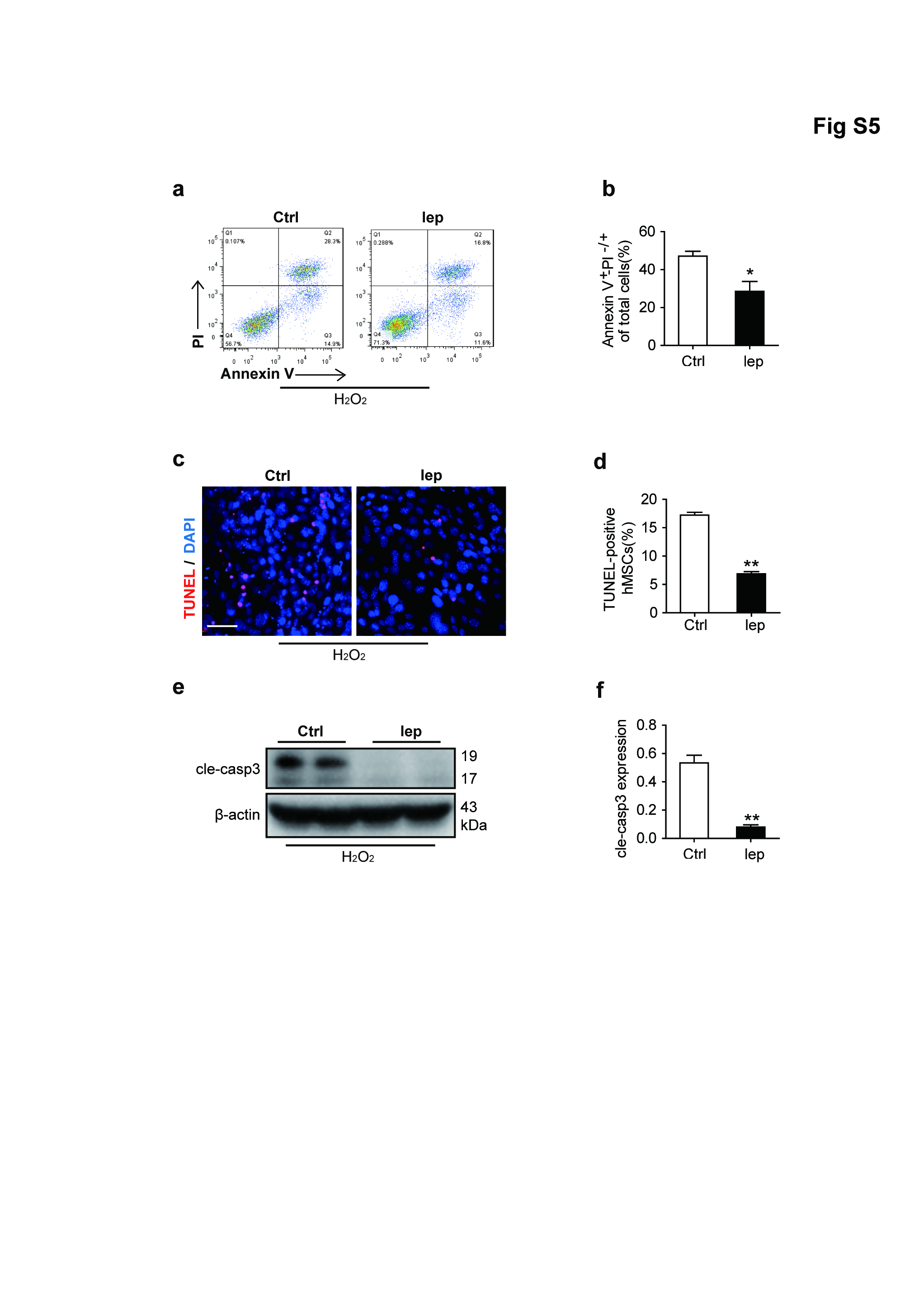

Supplement: Supplementary file 5 — Figure S5 [file 41419_2018_579_MOESM5_ESM.tif]

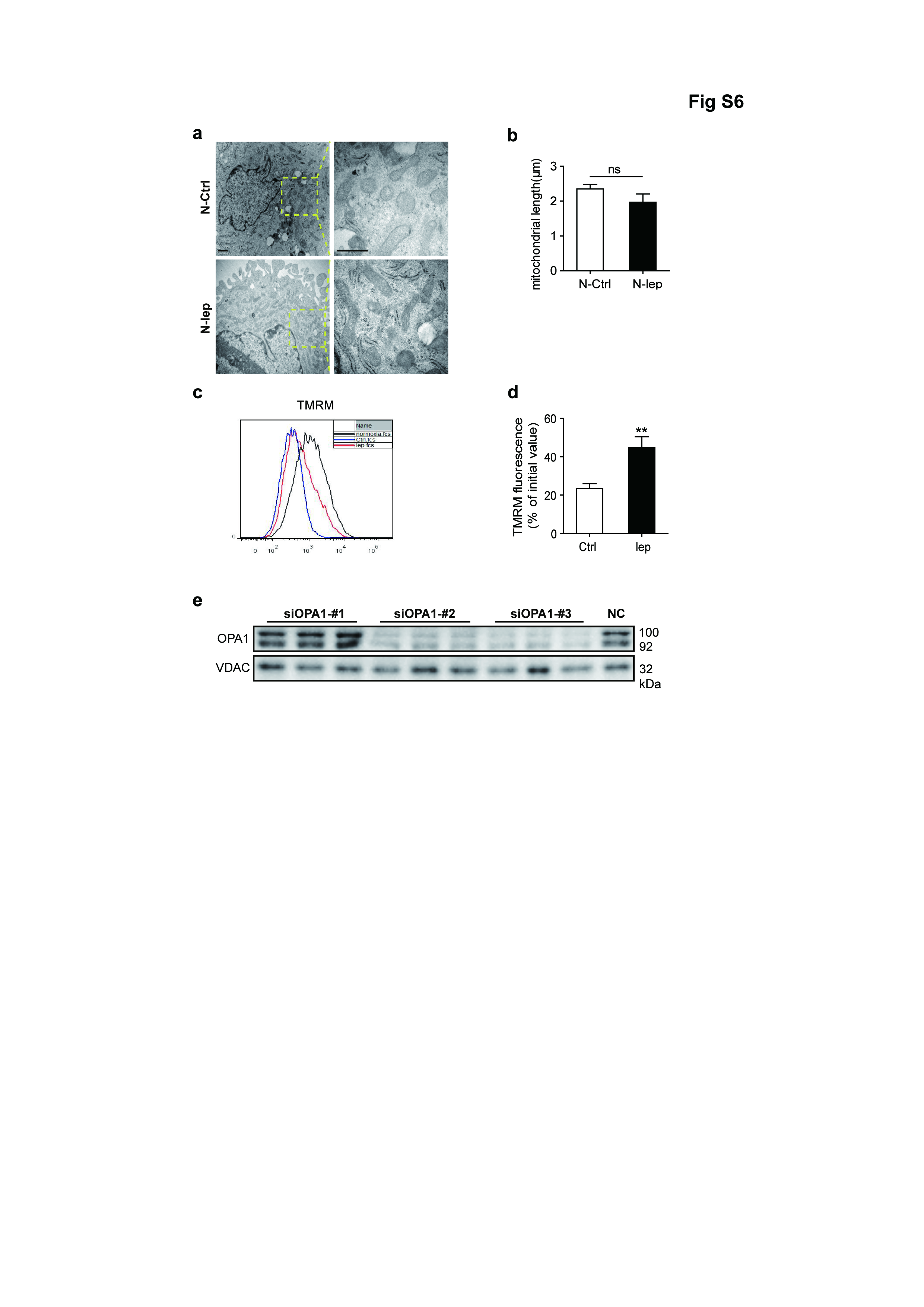

Supplement: Supplementary file 6 — Figure S6 [file 41419_2018_579_MOESM6_ESM.tif]

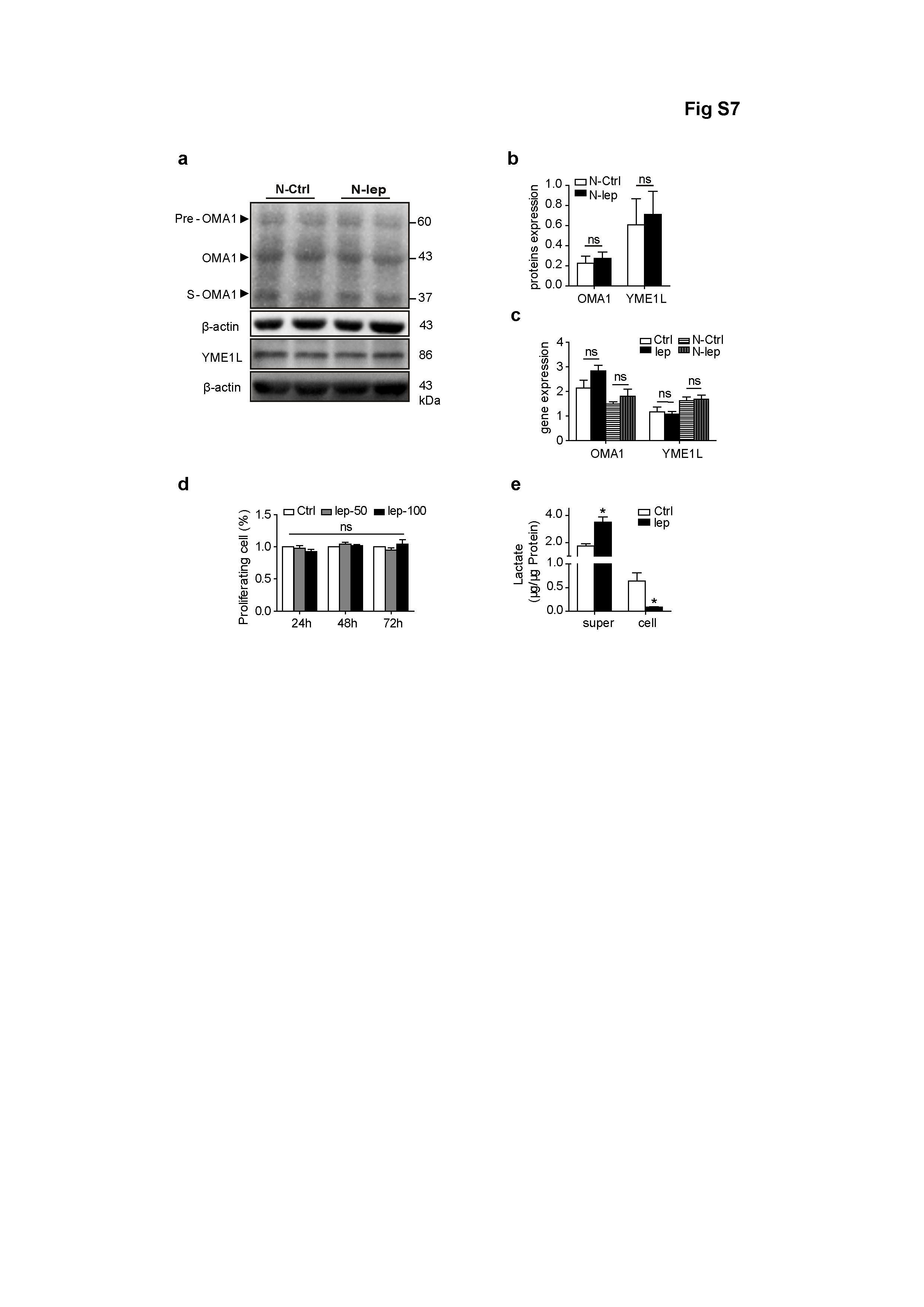

Supplement: Supplementary file 7 — Figure S7 [file 41419_2018_579_MOESM7_ESM.tif]

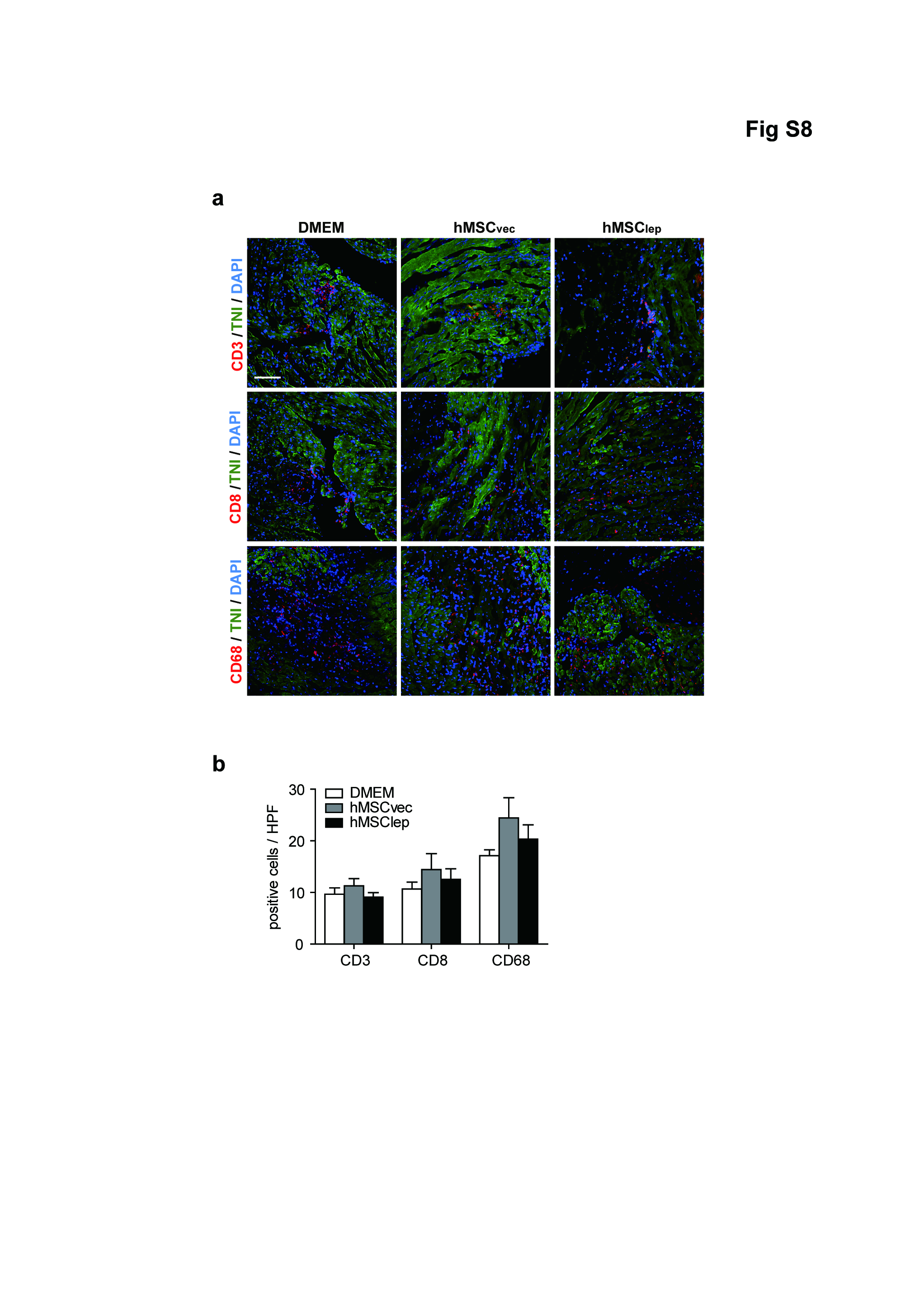

Supplement: Supplementary file 8 — Figure S8 [file 41419_2018_579_MOESM8_ESM.tif]
