# Supplementary material for: Leptin increases mitochondrial OPA1 via GSK3-mediated OMA1 ubiquitination to enhance therapeutic effects of mesenchymal stem cell transplantation
Source: Cell Death Dis. 2018 May 10;9(5):556. doi: 10.1038/s41419-018-0579-9 (PMC5945599; doi:10.1038/s41419-018-0579-9)
Supplement: Supplementary file 9 — Table S1 [file 41419_2018_579_MOESM9_ESM.docx]

Table S1. The table showed actual data of cardiac-function for each group at 28 day post-MI.

| **TABLE S1** | **DMEM-28d** | **Vec-28d** | **Leptin-28d** |
| --- | --- | --- | --- |
| EF(%) | 39.9±6.0 | 36.3 ±4.2 | 59.7±4.9 |
| FS(%) | 19.5 ±3.3 | 17.4±2.2 | 31.8±3.4 |
| LVEDD(mm) | 4.2±0.3 | 4.5±0.4 | 3.8±0.2 |
| LVESD(mm) | 3.4±0.3 | 3.8±0.4 | 2.6±0.2 |

Data were shown as mean ± SEM
