# Supplementary material for: Leptin increases mitochondrial OPA1 via GSK3-mediated OMA1 ubiquitination to enhance therapeutic effects of mesenchymal stem cell transplantation
Source: Cell Death Dis. 2018 May 10;9(5):556. doi: 10.1038/s41419-018-0579-9 (PMC5945599; doi:10.1038/s41419-018-0579-9)
Supplement: Supplementary file 10 — Table S2 [file 41419_2018_579_MOESM10_ESM.docx]

Table S2. Human primers for real-time PCR

| **Gene** | **Primer sequence 5`-3`** |
| --- | --- |
|  | **Forward Reverse** |
| *Drp1* CGTCGTAGTGGGAACGCAGA GCTTCCACCCCATTTTCTTCTCC  *Fis* CCGGACTCATCGGACTTGCT TGATGGGGCTGAAGGACGAA  *PGC1α* CACTCTCAGTAAGGGGCTGGT AACCAGAGCAGCACACTCGAT  *TFAM* GCTTATAGGGCGGAGTGGCA CAGCTTTTCCTGCGGTGAATC  *ATG5* AAGGCACACCACTGAAATGGC TGCAATCCCATCCAGAGTTGCT  *Mfn1* TGCCTCCTCTCCGCCTTTAAC CGCCTTCTTAGCCAGCACAA  *Mfn2* GAGGCGTAAGGAGTAGGCGG TCACTACGTCACCAGCGAGC  *OPA1* CTAGGATCGGCTGTTGGGGG GGTCTTCTGAACTAGGAAGGGCT  *OMA1* AAATTGGAGGCCGAAGCTGA TTGCCATGAGAAGGGTGTGT  *YME1L* CCAGCAGTGAGCCTTCACTTA GTCGATACAAGCAAGAGGAACG  *β-actin* CTCGCCTTTGCCGATCC GAATCCTTCTGACCCATGCC | |
