# Supplementary material for: Leptin increases mitochondrial OPA1 via GSK3-mediated OMA1 ubiquitination to enhance therapeutic effects of mesenchymal stem cell transplantation
Source: Cell Death Dis. 2018 May 10;9(5):556. doi: 10.1038/s41419-018-0579-9 (PMC5945599; doi:10.1038/s41419-018-0579-9)
Supplement: Supplementary file 11 — Table S3 [file 41419_2018_579_MOESM11_ESM.docx]

Table S3. Sense and antisense sequences of siRNA specific for OPA1

|  | **Target sequences of siRNAs** | |
| --- | --- | --- |
| **Gene** | **Sence** | **Antisence** |
| *OPA1(#1)* | GCCUGACAUUGUGUGGGAATT | UUCCCACACAAUGUCAGGCTT |
| *OPA1(#2)* | CCAUGUGGCCCUAUUUAAATT | UUUAAAUAGGGCCACAUGGTT |
| *OPA1(#3)* | GCAUGGCUCCUGACACAAATT | UUUGUGUCAGGAGCCAUGCTT |
