# Supplementary material for: Leptin increases mitochondrial OPA1 via GSK3-mediated OMA1 ubiquitination to enhance therapeutic effects of mesenchymal stem cell transplantation
Source: Cell Death Dis. 2018 May 10;9(5):556. doi: 10.1038/s41419-018-0579-9 (PMC5945599; doi:10.1038/s41419-018-0579-9)
Supplement: Supplementary file 12 — Supplemental information [file 41419_2018_579_MOESM12_ESM.docx]

**SUPPLEMENTAL INFORMATION:**

**Author contributions**:

JW, JH: Conception and design, financial support, being accountable for all aspects of the work.

XH, WZ, HY, JC, RW, XL: Conception and design, manuscript revising.

FY: Experiments performing, analyzing the data, assembled the figures and writing the manuscript.

RW, ZJ: Conception and design, manuscript revising, analyzing the data.

SS, WH, ZZ: Experiments performing.

CW, JN, NZ，KZ: Directing Experiments.

JZ, CN: Helping to measure and analyze cardiac function by echocardiography.

YW: Operating mice model of Myocardium Infarct Model.

**Antibodies:**

The following primary antibodies to proteins:

*For western blot:*

leptin (1:500, Abcam, Cambridge, MA, USA); leptin receptor (1:1000, Abcam, Cambridge, MA, USA); Mfn1 (1:1000, Cell Signaling Technology, Danvers, MA, USA); Mfn2 (1:1000, Cell Signaling Technology, Danvers, MA, USA); Drp1 (1:1000, Cell Signaling Technology, Danvers, MA, USA); PGC-1α (1:1000, Abcam, Cambridge, MA, USA); mono-OPA1 (1:1000, Abcam, Cambridge, MA, USA); poly-OPA1 (1:1000, BD Bioscience, San Jose, CA, USA); OMA1 (1: 300, Santa Cruz Biotechnology, Clinisciences, Naterre, France); YME1L (1:1000, Abcam, Cambridge, MA, USA); cleaved caspase3 (1:500, Cell Signaling Technology, Danvers, MA, USA); GSK3 (1:1000, Cell Signaling Technology, Danvers, MA, USA); phospho-GSK3 (1:1000, Abcam, Cambridge, MA, USA); Monoclonal anti-flag M2 antibody (1:1000, Sigma, Aldrich); HA-tag (1:1000, Cell Signaling Technology, Danvers, MA, USA); β-actin (1:3000, KANGCHEN, Shanghai, China); anti-rabbit, anti-goat and anti-mouse secondary antibody (1:5000, Abcam, Cambridge, MA, USA)

*For immunofluorescence:*

GFP primary antibody (1:400, Abcam, Cambridge, MA, USA); α-SMA (1:400, Abcam, Cambridge, MA, USA); CD31 (1:200, BD Bioscience, San Jose, CA, USA); vWF (1:50, Abcam, Cambridge, MA, USA); CD3 (1:200, Abcam, Cambridge, MA, USA); CD8 (1:200, Abcam, Cambridge, MA, USA); CD68 (1:200, BD Bioscience, San Jose, CA, USA); Cardiac Troponin I (1:200, Abcam, Cambridge, MA, USA); anti-rabbit, anti-goat and anti-mouse secondary antibody (1:400, Abcam, Cambridge, MA, USA)
