# Supplementary material for: Leptin increases mitochondrial OPA1 via GSK3-mediated OMA1 ubiquitination to enhance therapeutic effects of mesenchymal stem cell transplantation
Source: Cell Death Dis. 2018 May 10;9(5):556. doi: 10.1038/s41419-018-0579-9 (PMC5945599; doi:10.1038/s41419-018-0579-9)
Supplement: Supplementary file 13 — Supplementary figure legends [file 41419_2018_579_MOESM13_ESM.docx]

Supplementary figure legends

**Fig S1.** **Experiments of implanted hMSCs both *in vivo* and *in vitro***.

**Fig S2. Characteristics of hMSCs are described, and leptin receptor exists in hMSCs.** *(a)* Characteristics of hMSCs are described. Specific surface antigen markers of hMSCs, including allophycocyanin-CD90 (99.8%), PE-CD29 (99.6%), and PE-CD105 (99.6%), and negative surface antibodies such as PE-CD31 (0.1%), PE-CD34 (0%), and PE-CD117 (2.7%) were used to identify the characteristics of hMSCs via flow cytometry. *(b)* Leptin receptor (ObR) was identified in hMSCs by Western blot.

**Fig S3. Elevated expression level of leptin was confirmed in hMSCs.** *(a,b)* hMSCs were infected by leptin-overexpression lentivirus or viral vectors at MOI of 50, and efficiency of infection was measured by Western blot and immunofluorescence staining targeting leptin.

**Fig S4.** **In GSDH-induced apoptosis, similar protective effects of leptin on hMSCs were detected by leptin pretreatment or leptin overexpression *in vitro*.** *(a)* Annexin V/PI staining was analyzed among four groups: solvent-pretreated hMSCs (Ctrl), leptin-pretreated hMSCs (lep), vector-infected hMSCs (hMSC_vec_), and lentivirus-infected hMSCs (hMSC_lep_). *(b)* Early (Q3) and late (Q2) apoptotic rates were evaluated. *(c)* Representative TUNEL and DAPI staining images were captured at 400× (Scale bar, 50 μm). *(d)* The corresponding chart of TUNEL staining was quantified for TUNEL-positive nuclei with respect to total nuclei. *(e,f)* Cleaved caspase 3 protein expression levels of WCL were assessed by Western blot in the above four groups; β-actin served as a control, and protein expression levels were quantified by densitometry. Each *in vitro* experiment was repeated three times. Data were shown as mean ± SEM. **denotes *P*<0.01.

**Fig S5. Leptin improved hMSCs survival response to H_2_O_2_-induced apoptosis.** *(a)* Apoptosis examination was shown by Annexin V/PI staining and *(b)* early (Q3) and late (Q2) apoptotic rates were enumerated. *(c,d)* Representative images of TUNEL and DAPI staining (Scale bar, 50 μm) and quantification of TUNEL-positive nuclei over the total nuclei were shown. *(e,f)* The expression of cleaved caspase 3 protein was tested by Western blot, normalized to β-actin. The protein level was quantified by densitometry analysis. Three independent experiments were repeated. Data were shown as mean ± SEM. *denotes *P*<0.05, ***P*<0.01.

**Fig S6.** **Administration of leptin did not influence mitochondria under physiological condition.** *(a)* Mitochondrial ultrastructures were analyzed by TEM in hMSCs-Lep^pre^ and hMSCs-Ctrl^pre^ under normoxia culture condition (magnification was set at 15,000× and 50,000×, respectively). Scale bar, 1 μm. *(b)* Mitochondrial length was measured at least 20 mitochondria for each cell (at least 30 cells for each group). *(c,d)* After hMSCs incubated with TMRM (50 nM) for 30 min, mitochondrial membrane potential (ψMt) was measured using flow cytometry and quantified by analysis of mean of fluorescence intensity in hMSCs-Ctrl^pre^ and hMSCs-Lep^pre^ groups and normal cultured hMSCs. *(e)* Verification of OPA1 knockdown efficiency using siRNA was proved via Western blot. Three independent experiments were repeated. Data were shown as mean ± SEM. *denotes *P*<0.05, ***P*<0.01.

**Fig S7.** **Effect of leptin had no impact on OMA1 and YME1L under physiological condition.** *(a)* Immunoblot analysis of OMA1 and YME1L proteins expression in hMSCs-Lep^pre^ or hMSCs-Ctrl^pre^ groups under normoxia. *(b)* Quantification of OMA1 and YME1L protein levels were normalized by β-actin. *(c)* OMA1 and YME1L mRNA expression levels were presented relative to *β-actin* in both normal condition and GSDH stress. *(d)* Cells proliferation was assessed using CCK 8 at indicated time points after administration of leptin at 50 or 100 ng/ml under normoxia. *(e)* Lactate production of supernatant or cellular was measured in hMSCs-Ctrl^pre^ and hMSCs-Lep^pre^, respectively, under GSDH stress for 24 h. Three independent experiments were repeated. Data were shown as mean ± SEM. *denotes *P*<0.05, ***P*<0.01.

**Fig S8. Transplanted hMSCs did not induce significant change in immunoreactions and inflammatory response.** *(a)* Inflammatory response were assessed by immunostaining for expression of CD3, CD8 and CD68 in peri-infarcted zone in DMEM, hMSC_vec_ and hMSC_lep_ groups at day 3 post-MI *in vivo* (n=3 for each group). Scale bar, 100 μm.*（b）*CD3, CD8 and CD68 stained positiive cells were countered by 6-8 HPF per section in the bar graphs. Three independent experiments were repeated. Data were shown as mean ± SEM. *denotes *P*<0.05, ***P*<0.01.
